# A meta-analysis of idiopathic granulomatous mastitis treatments for remission and recurrence prevention

**DOI:** 10.3389/fmed.2024.1346790

**Published:** 2024-05-30

**Authors:** Seeu Si Ong, Peh Joo Ho, Jonathan Jun Kit Liow, Qing Ting Tan, Serene Si Ning Goh, Jingmei Li, Mikael Hartman

**Affiliations:** ^1^Genome Institute of Singapore (GIS), Agency for Science, Technology and Research (A*STAR), Singapore, Singapore; ^2^Department of Surgery, Yong Loo Lin School of Medicine, National University of Singapore, Singapore, Singapore; ^3^Saw Swee Hock School of Public Health, National University of Singapore, Singapore, Singapore; ^4^KK Breast Department, KK Women’s and Children’s Hospital, Singapore, Singapore; ^5^Department of Surgery, University Surgical Cluster, National University Health System, Singapore, Singapore

**Keywords:** idiopathic granulomatous mastitis, meta-analysis, remission, recurrence prevention, treatment efficacy, follow-up duration, evidence-based guidelines, IGM

## Abstract

**Purpose:**

The major aim of our meta-analysis was to review the effectiveness of various treatment modalities for achieving successful remission and preventing recurrence for women with idiopathic granulomatous mastitis (IGM). This knowledge is instrumental in developing evidence-based guidelines for clinicians to improve management strategies and outcomes for patients with IGM.

**Methods:**

A systematic literature search was performed on MEDLINE (Ovid), Embase (Elsevier), PubMed, Cochrane Library, Web of Science, and Google Scholar; studies published to 19 January 2022 were included. A meta-analysis of 57 observational studies was performed. The results of two randomized controlled trials were also examined.

**Results:**

There were 3,035 IGM patients across the observational and randomised studies. Overall recurrence and remission rates across all treatment strategies in 59 studies are 87.9% (2,667/3035) and 13.5% (359/2667), respectively. The studies reported 19 different treatment strategies, comprising observation, medical monotherapies, surgery, and combinations involving medical therapies, with and without surgery. Among monotherapy treatment, surgical management had the highest pooled remission rate (0.99 [95% confidence interval (CI) = 0.97–1.00]); among combination therapy, this was steroids and surgery (0.99 [0.94–1.00]). Antibiotic monotherapy had the lowest remission rate (0.72 [0.37–0.96]). The highest recurrence rates belonged to treatments that combined antibiotics and surgery (0.54 [0.02–1.00]), and antibiotics, steroids, and surgery (0.57 [0.00–1.00]). Most successful for preventing recurrence were observation (0.03 [0.00–0.10]), methotrexate (0.08 [0.00–0.24]), and steroids and surgery (0.05 [0.01–0.12]). There is a significant association between longer follow-up duration and recurrence rate reported, *p* = 0.002.

**Conclusion:**

Combination therapies, especially those incorporating antibiotics, steroids, and surgery, have demonstrated higher remission rates, challenging the use of antibiotic monotherapy. There is an increased emphasis on the need for personalised, multi-pronged approach for preventing IGM recurrence, with longer follow-up care. More prospective future work in IGM research, with standardised diagnostic criteria, treatment protocols, and reporting guidelines will be important for developing treatment protocols and guidelines clinicians can adhere to in the clinical management of IGM patients.

**Systematic review registration**: PROSPERO (CRD42022301386).

## 1 Introduction

Idiopathic granulomatous mastitis (IGM) is a rare and perplexing breast inflammation (1). The absence of an obvious aetiology poses significant challenges for achieving remission and preventing recurrence (2). While various treatment strategies have been explored for managing IGM, the inflammatory nature of the disease and its ambiguous aetiology make for complex disease management strategies (3). Surgical approaches of invasive excisions to remove affected breast tissue were originally the common treatment strategy for managing IGM (4); surgery has also been used to manage recurrent cases (5). Medical management gained prominence since IGM presents clinically similar to breast abscess, leading clinicians to treat IGM patients with antibiotics (6). Other medical management approaches, such as corticosteroids, and immunosuppressants, like methotrexate, were thought to control inflammation and reduce the need for invasive procedures for IGM patients (7, 8) Observation, with clinical follow-up, has also been recommended (7).

The long natural history and recurrence of the disease complicate decision-making for treatment allocation and follow-up (9). There has been a shift towards combination medical therapies, with or without surgery, to manage the long-course and recurrent cases (10). Unfortunately, individual studies investigating the efficacy of treatment strategies, as monotherapies, or combination therapies, are often limited in statistical power given the rarity of IGM (11). Few studies have sufficient patient follow-up for evaluating recurrence prevention (12–14). As a result, clinical practice for managing IGM differs across treatment centres and among clinicians, largely based on anecdotal experience and personal preference (3, 15, 16). There is a need for evidence-based and universally adhered-to treatment guidelines for managing IGM (9).

Existing work to synthesise published studies is outdated (17) or restricted to comparisons between treatment sub-groups (15, 16). Our systematic review and meta-analysis of published literature will provide insights into the effectiveness across various treatment modalities for achieving remission and preventing recurrence in patients with IGM. Specifically, this study investigates the remission rates and recurrence prevention outcomes associated with different interventions, including medical therapies such as antibiotics, corticosteroids, immunosuppressive agents, and surgical interventions such as abscess drainage, excision, and mastectomy. This study also describes the remission categorisation, follow-up duration, and geographical spread of the studies included.

Our study aims to contribute to a comprehensive understanding of the efficacy of different treatment options. This knowledge will be instrumental in developing evidence-based guidelines for clinicians, potentially improving management strategies and outcomes for patients with IGM.

## 2 Materials and methods

The systematic review and meta-analysis followed the Preferred Reporting Items for Systematic Reviews and Meta-Analyses (PRISMA) guidelines (18). The study was registered on PROSPERO (CRD42022301386) prior to commencement. The study did not require institutional review board approval.

### 2.1 Literature search

A systematic literature search was performed on MEDLINE (Ovid), Embase (Elsevier), PubMed, Cochrane Library, Web of Science, and Google Scholar. The search strategy involved combining disease (idiopathic granulomatous mastitis), treatment (antibiotics, steroids, methotrexate, surgery, or observation), and outcome measures of interest (remission, and recurrence). Each database was searched with its corresponding keyword matching, title, and abstract search. The full search strategy can be found in Appendix A. The reference list of included studies and relevant systematic reviews were manually searched.

### 2.2 Study selection

Studies published up until 19 January 2022 were included. EndNote (version 20.4, Bld 18,004) was used to remove duplicate records.

I Patient

Studies with IGM patients were included. We only included studies that confirm IGM diagnosis with histopathology and exclude tuberculosis and fungal causes. Studies exclusively with non-IGM patients, with breastfeeding and/or pregnant patients, or studies without the IGM diagnosis confirmation described above, were excluded.

II Intervention

Studies reporting the following treatment were included: Antibiotics, corticosteroids, including but not limited to topical and oral (systemic) forms of medication, methotrexate, any surgical management, including but not limited to mastectomy, lumpectomy, excisions, and incision and drainage, and observation. Studies reporting other treatment types, including but not limited to anti-tuberculosis medication, traditional Chinese medication, and alternative treatments were excluded.

III Outcome to measure

Studies reporting complete remission rate and recurrence rate were included. Studies that did not report remission and/or recurrence rates, or with missing information in those measurements, were excluded. Studies without treatment-specific remission and/or recurrence rates were also excluded.

IV Study design

Observational studies and randomised controlled trials (RCT) were included. Non-original research articles, non-English publications, reviews, editorial articles, conference abstracts, letters and commentaries, clinical trial registrations, or case reports were excluded.

EndNote (version 20, Bld 18,004) was used to detect duplicated entries and perform the title and abstract screen. After the initial title and abstract screen was performed to exclude articles not relevant to the review, two independent reviewers (SSO and JJKL) performed full-text review as per the inclusion and exclusion criteria described above, with 95.2% consensus. Disagreements over study inclusion were resolved by discussion. Data extraction was also performed by the two independent reviewers (SSO and JJKL) using Excel [version 16.77.1, (23091703)]. The predefined data extraction fields, included recruitment years, case numbers, mean and/or median age of patients, treatment type, complete remission definition, number of patients achieving complete remission, number of patients subsequently recurred and follow-up duration. Disagreements over data extracted were resolved by discussion, as well as, seeking expert opinion.

### 2.3 Quality assessment

Study quality assessment for observational studies was assessed with the 9-star Newcastle–Ottawa Scale (NOS), based on selection (4 stars), comparability (2 stars), and outcome (3 stars) (19, 20). NOS scores for the observational studies were converted to Agency for Healthcare Research and Quality (AHRQ, United States) standards with the following thresholds (21):

- Good quality: 3–4 stars from selection, 1–2 stars from comparability, and 2–3 stars from outcome
- Fair quality: 2 stars from selection, 1–2 stars from comparability, and 2–3 stars from outcome
- Poor quality: 0–1 star(s) from selection, 0 stars from comparability, or 0–1 stars from outcome

Study quality assessment for RCTs was assessed with the 5-point Jadad score. Points were awarded if the study was described as randomised, randomisation was described as performed with appropriate methods, the study was double-blinded for participants and treatment administrators, double-blinding was with appropriate methods, and withdrawals and dropouts were sufficiently described (22). A point was deducted each if the randomisation or double-blinding method described was inappropriate (22). Jadad scores threshold conversion was defined as

- Good quality: Appropriate randomisation and blinding (4–5 points)
- Fair quality: Appropriate randomisation and inappropriate/absent blinding (2–3 points)
- Poor quality: Inappropriate/absent randomisation and blinding (0–1 point(s))

### 2.4 Statistical analysis

For the observational studies, remission rates and recurrence rates were calculated, and arcsine transformed as a variance stabilisation transformation (23). Random-effects meta-analysis model was used to estimate the pooled remission and recurrence rates using *rma* (*metafor* package, version 4.4.0) (24). Sensitivity analyses were performed for individual treatment modalities:

- Medication monotherapy: Antibiotics only, peroral corticosteroids only, corticosteroids only (peroral, topical and injected), and methotrexate only
- Combination therapy: Combination therapy including antibiotics, combination therapy including corticosteroids, and combination therapy including methotrexate
- Medication without surgery
- Surgery, with and without medication: Biopsy and aspiration only, excisional surgery
- All other combinations of medication and surgery
- Observation only

Forest plots were generated to visualise the remission and recurrence rates and their confidence intervals of individual studies, and the estimated pooled remission and recurrence proportions. Random-effects model was selected since study populations and sizes were highly varied, and remission and recurrence rates were likely to be highly varied across studies as well. 95% confidence intervals (95%CI), z-score and the *p*-value for the overall effect were calculated. Statistical heterogeneity was estimated with the restricted maximum likelihood estimator (method = “REML”) (24, 25), using the I^2^ and τ^2^ statistics.

Linear regression models were used to estimate the regression coefficient and *p*-value for the association between recurrence rate and follow-up duration. Residuals were inspected. Publication bias across the different treatment types was assessed with Begg’s rank correlation test and visualised with funnel plots (24, 26).

All analyses were performed with R (version 4.0.4) unless otherwise stated. The systematic review and meta-analysis used published data and did not involve direct patient contact.

## 3 Results

### 3.1 Search results

**Figure 1:**
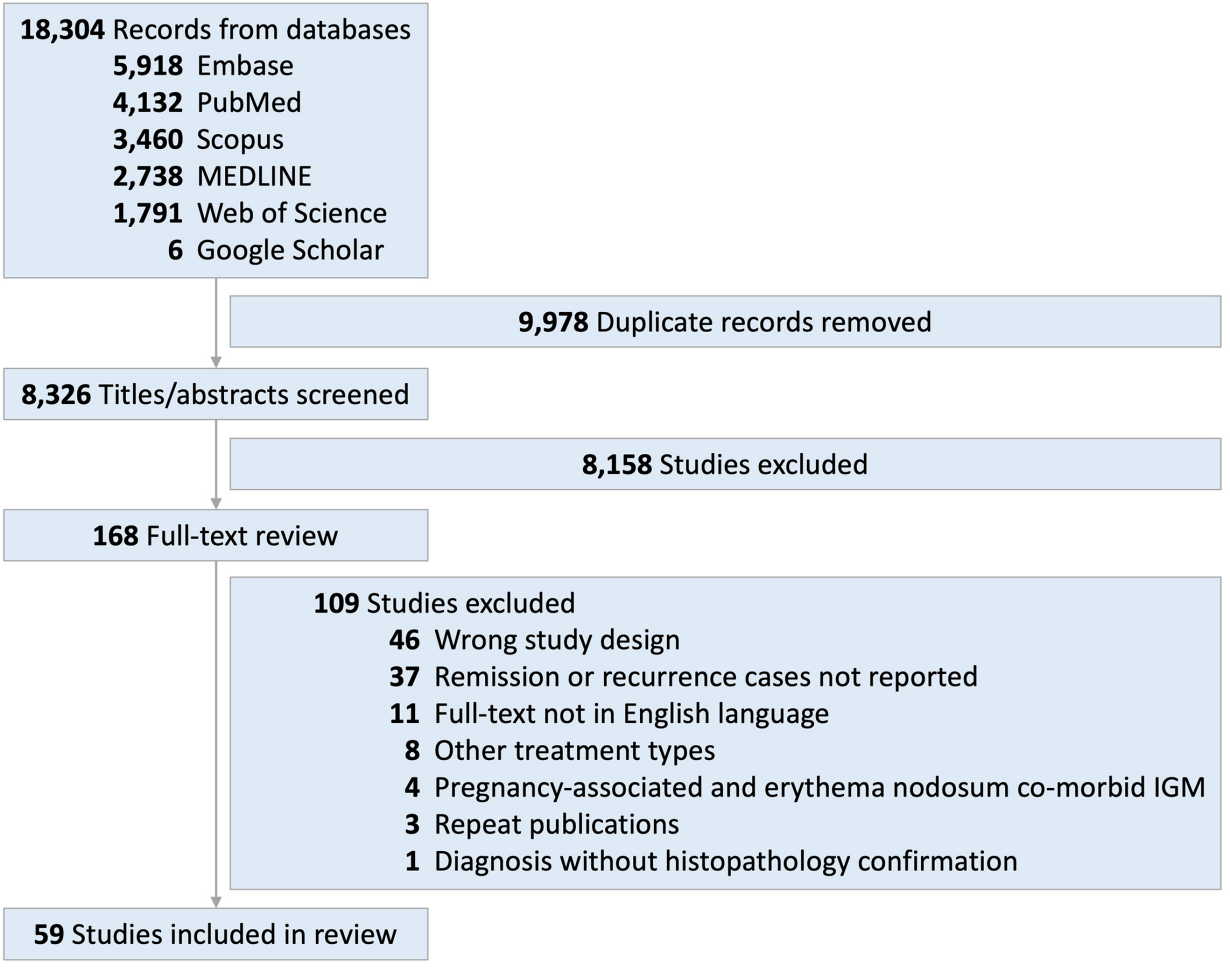
Flowchart of articles selected to be included in meta-analysis.

The systematic search strategy yielded 18,304 references. Full-text review was performed for 168 records, by the two independent reviewers, and 59 articles were included in this systematic review (7, 8, 27–83). The flowchart of the search results and included studies is displayed in Figure 1. Manual search of reference lists did not yield additional articles.

### 3.2 Studies characteristics

Of the 59 articles included in the review, 57 (96.6%) articles were non-randomised observational studies and only 2 (3.4%) articles were RCTs. Observational studies were retrospective (44/57), prospective (11/57), and unspecified (2/57; Table 1). There were 3,035 IGM patients across the randomised (258/3035, 8.5%) and observational studies (2,777/3035, 91.5%). Overall recurrence and remission rates across all treatment strategies in 59 studies are 87.9% (2,667/3035) and 13.5% (359/2667), respectively.

**Table 1:** Studies included in the meta-analysis. n: sample size of patients, reported in the study.

Randomised studies							
Source	Treatment strategy	n	Patients achieved remission (%)	Patients with recurrence (%)	Follow-up duration (months)	Quality assessment category	Country
Mawla et al., 2020 (27)	Steroids, and surgery	150	140 (93.3%)	10 (7.1%)	18	Fair	Egypt
Çetin et al., 2019 (28)	Topical steroids, peroral steroids, and combined topical and peroral steroids	108	90 (83.3%)	17 (18.9%)	21.8	Fair	Turkey
Non-randomised studies							
Wang et al., 2021 (29)	Steroids, and surgery	356	200 (56.2%)	18 (9%)	15.6	Good	China
Zhang et al., 2019 (30)	Surgery	164	164 (100%)	8 (4.9%)	29	Good	China
Davis et al., 2019 (31)	Observation, and surgery	118	118 (100%)	19 (16.1%)	Not available	Good	USA
Çetinkaya et al., 2021 (32)	Observation, antibiotics, steroids, and surgery	116	116 (100%)	14 (12.1%)	58	Fair	Turkey
Shojaee et al., 2021 (33)	Steroids, and surgery	87	87 (100%)	25 (28.7%)	26	Good	Iran
Ertürk et al., 2021 (34)	Injected steroids, and surgery	86	84 (97.7%)	15 (17.9%)	12	Good	Turkey
Tan, et al., 2019 (35)	Observation, antibiotics, steroids, and methotrexate	78	67 (85.9%)	20 (29.9%)	8.4	Good	Singapore
Toktas et al., 2021 (36)	Injected steroids, topical steroids, and peroral steroids	78	63 (80.8%)	19 (30.2%)	20.35	Good	Turkey
Yabanoğlu et al., 2015 (37)	Steroids, surgery	77	77 (100%)	9 (11.7%)	16.57	Poor	Turkey
Akcan et al., 2014 (8)	Antibiotics, steroids, and surgery	74	74 (100%)	4 (5.4%)	41	Good	Turkey
Wang et al., 2020 (38)	Steroids, and surgery	69	69 (100%)	7 (10.1%)	13	Good	China
Zhang et al., 2020 (39)	Surgery	68	68 (100%)	3 (4.4%)	24	Good	China
Deng et al., 2017 (40)	Steroids, and surgery	65	53 (81.5%)	12 (22.6%)	12	Poor	China
Altunkeser et al., 2019 (41)	Antibiotics, steroids, and surgery	62	45 (72.6%)	1 (2.2%)	27.84	Good	Turkey
Dalbasi et al., 2021 (42)	Steroids, surgery, and methotrexate	62	62 (100%)	7 (11.3%)	24	Good	Turkey
Karanlik et al., 2014 (43)	Steroids, and surgery	60	60 (100%)	7 (11.7%)	25	Good	Turkey
Kundaktepe et al., 2021 (44)	Methotrexate	60	60 (100%)	8 (13.3%)	27.7	Fair	Turkey
Cornejo-Juárez et al., 2014 (45)	Observation, antibiotics, steroids, and surgery	58	43 (74.1%)	0 (0%)	16.7	Good	Mexico
Atalay et al., 2011 (46)	Surgery	51	51 (100%)	3 (5.9%)	38	Poor	Turkey
Fayed et al., 2019 (47)	Steroids, and surgery	50	50 (100%)	2 (4%)	12	Good	Egypt
Tekgöz et al., 2020 (48)	Observation, steroids, surgery, and methotrexate	50	42 (84%)	8 (19%)	13.83	Good	Turkey
Hur et al., 2013 (49)	Observation, antibiotics, steroids, and surgery	49	27 (55.1%)	1 (3.7%)	32	Good	Korea
Pandey et al., 2014 (50)	Observation, steroids, and surgery	49	40 (81.6%)	10 (25%)	9	Good	USA
Sen Oran et al., 2013 (51)	Steroids, and surgery	46	43 (93.5%)	8 (18.6%)	35.4	Good	Turkey
Atak et al., 2015 (52)	Antibiotics, steroids, and surgery	40	40 (100%)	15 (37.5%)	24.85	Fair	Turkey
Oak et al., 2021 (53)	Steroids, and methotrexate	40	40 (100%)	2 (5%)	12	Good	India
Chirappapha et al., 2018 (54)	Steroids, and surgery	36	24 (66.7%)	5 (20.8%)	20.73	Poor	Thailand
Shin et al., 2017 (55)	Antibiotics, steroids, and surgery	36	31 (86.1%)	6 (19.4%)	45.5	Good	Korea
Chen et al., 2021 (56)	Steroids, surgery, and methotrexate	35	31 (88.6%)	3 (9.7%)	29.5	Good	China
Bouton et al., 2015 (57)	Observation, and surgery	32	32 (100%)	3 (9.4%)	Not available	Poor	USA
Elzahaby et al., 2016 (58)	Surgery	30	30 (100%)	1 (3.3%)	19	Good	Egypt
Farouk et al., 2017 (59)	Antibiotics	30	30 (100%)	0 (0%)	15.5	Good	Egypt
Kayahan et al., 2012 (60)	Steroids, and surgery	30	26 (86.7%)	5 (19.2%)	28.7	Poor	Turkey
Liao et al., 2020 (61)	Surgery	30	29 (96.7%)	3 (10.3%)	12	Fair	China
Maher et al., 2021 (62)	Steroids, and surgery	30	28 (93.3%)	6 (21.4%)	Not available	Poor	Egypt
Alrayes et al., 2019 (63)	Antibiotics, and surgery	29	29 (100%)	0 (0%)	18	Fair	Bahrain
Alper et al., 2020 (64)	Injected steroids	28	26 (92.9%)	0 (0%)	11.8	Poor	Turkey
Altintoprak et al., 2015 (65)	Topical steroids	28	28 (100%)	3 (10.7%)	37.2	Good	Turkey
Aldaqal, et al., 2004 (66)	Observation, antibiotics, steroids, and surgery	25	14 (56%)	4 (28.6%)	Not available	Poor	Saudi Arabia
Kiyak et al., 2014 (67)	Surgery	24	24 (100%)	2 (8.3%)	34.8	Poor	Turkey
Néel et al., 2013 (68)	Antibiotics, steroids, surgery, and methotrexate	21	19 (90.5%)	14 (73.7%)	72	Good	France
Gurleyik et al., 2012 (69)	Steroids, and surgery	19	19 (100%)	1 (5.3%)	20	Good	Turkey
Postolova et al., 2020 (70)	Methotrexate	19	12 (63.2%)	2 (16.7%)	36	Good	USA
Dağ et al., 2021 (71)	Surgery	18	18 (100%)	2 (11.1%)	20	Good	Turkey
Eser et al., 2013 (72)	Steroids, and surgery	17	17 (100%)	2 (11.8%)	36	Poor	Turkey
Kafadar et al., 2021 (7)	Methotrexate	17	10 (58.8%)	0 (0%)	6.5	Poor	Turkey
Ocal et al., 2010 (73)	Surgery	16	13 (81.2%)	3 (23.1%)	24	Good	Turkey
Joseph et al., 2014 (74)	Steroids	15	12 (80%)	7 (58.3%)	15	Fair	USA
Skandarajah et al., 2015 (75)	Observation, antibiotics, steroids, and surgery	14	12 (85.7%)	4 (33.3%)	6	Poor	Australia
Ahmed et al., 2016 (76)	Antibiotics, steroids, and surgery	13	13 (100%)	2 (15.4%)	24	Fair	Egypt
Çalış et al., 2014 (77)	Steroids	13	13 (100%)	2 (15.4%)	24	Good	Turkey
Akahane et al., 2013 (78)	Antibiotics, steroids, and surgery	12	12 (100%)	2 (16.7%)	22	Good	Japan
Erhan et al., 2000 (79)	Surgery	12	12 (100%)	2 (16.7%)	Not available	Poor	Turkey
Gunduz et al., 2014 (80)	Topical steroids	11	11 (100%)	2 (18.2%)	17	Good	Turkey
Lai et al., 2005 (81)	Observation, and surgery	9	5 (55.6%)	0 (0%)	18.7	Good	Hong Kong
Sakurai et al., 2011 (82)	Antibiotics, steroids, and surgery	9	8 (88.9%)	1 (12.5%)	40.3	Good	Japan
Berkesoglu et al., 2021 (83)	Antibiotics, steroids, surgery, and methotrexate	6	6 (100%)	0 (0%)	37.5	Fair	Turkey

The studies reported 19 different treatment strategies, comprising observation, medical monotherapies, surgery, and combinations involving medical therapies, with and without surgery. These treatment strategies are listed in Supplementary Table 1.

The studies were conducted across 16 countries, with nearly half the studies recruiting patients in Turkey (27 studies, 45.8%). The remaining countries are China (7 studies, 11.9%), Egypt (6 studies, 10.2%), United States of America (5 studies, 8.5%), Japan (2 studies, 3.4%), and Korea (2 studies, 3.4%); Australia, Bahrain, France, Hong Kong, India, Iran, Mexico, Saudi Arabia, Singapore, and Thailand had 1 study (1.7%) each.

Forty of the studies (67.8%) described their clinical and/or radiological definition for successful remission; this was not the case in the remaining 19 studies (32.2%). The median sample size across all studies was 36 [range = 6–356] patients; studies included a median of 2 [range = 1–8] treatment types. For each treatment strategy in observational studies, the median sample size was 13.5 [range = 1–200] patients (Supplementary Table 1). The median sample size for each treatment strategy in the RCTs was 41.0 [range = 33.0–75.0] patients.

### 3.3 Remission

**Figure 2:**
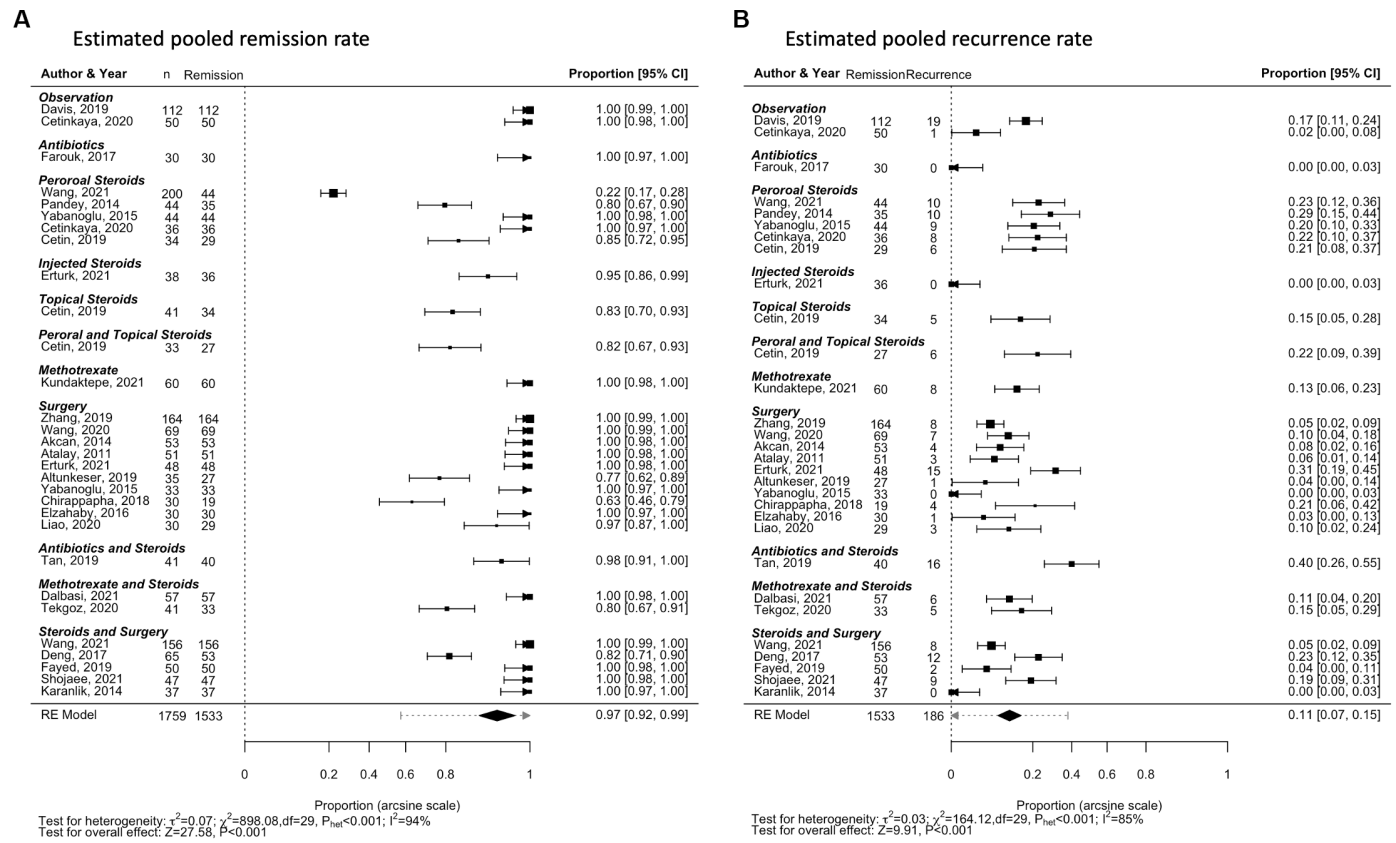
Forest plots for remission and recurrence rates of all treatment types, for treatment modalities reported with n ≥ 30. **(A)** Estimated pooled remission rate. **(B)** Estimated pooled recurrence rate.

Examining studies with treatment modalities of higher statistical power (*n* ≥ 30) among the 57 observational studies, a random-effects meta-analysis of pooled data from 24 studies (42.1%) found the estimated proportion of successful remission is 0.97 [95%CI 0.92–0.99] (Figure 2A) (8, 28–35, 37, 38, 40–44, 46–48, 50, 54, 58, 59, 61). The level of heterogeneity between studies was high, with an I^2^ statistic of 94.2% (*p*_het_ < 0.001; Figure 2A). The unadjusted pooled remission rate that includes studies with treatment modalities of lower statistical power (*n* < 30 IGM patients) was lower: 0.95 [0.92–0.97].

The pooled remission and recurrence rates estimates are also depicted for treatment strategies reported in more than 3 studies (Supplementary Table 1): Observation (Supplementary Figure 1), antibiotics monotherapy (Supplementary Figure 2), variations of steroid treatment (Supplementary Figure 3), methotrexate monotherapy (Supplementary Figure 4), surgical treatment (Supplementary Figure 5), and combination therapy (Supplementary Figure 6).

Examining individual treatment modalities found that the combination of antibiotics, steroids, and surgery had the highest random-effects meta-analysis estimate for remission at 1.00 [0.82–1.00, *p* < 0.001, I^2^ = 0%, *p*_het_ = 1.00] (Supplementary Table 1; Supplementary Figure 6G). However, this was only reported in 5 patients, across 3 studies (75, 82, 83). All surgery (0.99 [0.97–1.00]), excisional surgery (0.99 [0.96–1.00]), and combination therapy with steroids and surgery (0.99 [0.94–1.00]) were also found to have high pooled proportion for achieving remission (Supplementary Table 1; Supplementary Figures 5A,G, 6E). Heterogeneity among the pooled studies ranged from moderate to high: 73.6, 69.8, and 82.4%, respectively (Supplementary Table 1). Among treatment strategies reported in 4 or more studies, antibiotics monotherapy had the lowest estimate for remission: 0.72 [0.37–0.96] (*p* < 0.001, I^2^ = 92.6%, *p*_het_ < 0.001; Supplementary Table 1; Figure 2A). Study heterogeneity (I^2^) ranged from 68.8% (antibiotics and steroid combination therapy) to 93.9% (methotrexate monotherapy; Supplementary Table 1). Pooled estimates and the corresponding heterogeneity of studies for the remaining treatment modalities are summarised in Supplementary Table 1.

The two randomised studies compared different treatment modalities. The Cetin, et al., 2019 study comparison of topical steroids (37/41, 88.1%), peroral steroids (31/34, 73.8%), and a combination of the two variations (29/33, 72.5%) did not report significant differences in treatment response to achieve remission (*p* = 0.16) (28). Contrastingly, the Mawla, et al., 2020 study found significant differences comparing treatment response to surgical management (75/75, 100%) and steroid treatment (65/75, 86.7%) for achieving remission (*p* = 0.001) (27). Both studies also recorded and reported the recovery duration for the treatment modalities compared, with significantly shorter treatment duration for peroral steroid treatment (*p* < 0.001) as compared to topical steroid application and combination peroral and topical steroid treatment in the Cetin, 2019 study; recovery from surgical treatment was significantly shorter (*p* = 0.002) as compared to steroid treatment in the Mawla, 2020 study (27, 28).

### 3.4 Recurrence

The same 24 studies (42.1%) reporting treatment strategies with higher statistical power (*n* ≥ 30) found the estimated recurrence rate is 0.11 [0.07–0.15] (Figure 2B) (8, 28–35, 37, 38, 40–44, 46–48, 50, 54, 58, 59, 61). The unadjusted estimate that includes studies with n < 30 IGM patients was only slightly higher: 0.12 [0.08–0.15]. The level of heterogeneity between the higher-powered studies (n ≥ 30) was high, with an I^2^ statistic of 84.7% (*p*_het_ < 0.001; Figure 2B).

Examining individual treatment modalities found that observation had the lowest random-effects meta-analysis estimate for recurrence at 0.03 [0.00–0.10, *p* = 0.02, I^2^ = 56.2%, *p*_het_ = 0.01] (Supplementary Table 1; Figure 1B). This estimate pooled data from 10 studies; the median number of recurrence patients reported was 0 [range 0–119] (31, 32, 35, 45, 48–50, 57, 66, 81). Other treatment modalities with pooled recurrence proportion estimated at less than 0.10 were steroids and surgery combination therapy (0.05 [0.01–0.12]), all surgery with n ≥ 30 IGM patients recruited (0.08 [0.02–0.15]), methotrexate monotherapy (0.08 [0.00–0.24]), and antibiotics and steroids combination therapy (0.08 [0.00–0.30]; Supplementary Table 1; Supplementary Figures 4B, 5D, 6B,F). Heterogeneity among the pooled studies ranged from moderate to high: 78.3, 83.9, 64.3, and 84.1%, respectively (Supplementary Table 1). Excluding treatment strategies reported in less than three studies, antibiotics, steroids, and surgery combination therapy had the highest estimate for remission: 0.57 [0.00–1.00] (*p* = 0.03, I^2^ = 63.6%, *p*_het_ = 0.08; Supplementary Table 1; Supplementary Figure 6H). Study heterogeneity (I^2^) ranged from 56.2% (observation) to 89.6% (antibiotics and steroids). Pooled estimates and the corresponding heterogeneity of studies for the remaining treatment modalities are summarised in Supplementary Table 1.

In the randomised studies, the recurrence rate was not significantly different (*p* = 0.54) comparing topical, peroral and combination steroid treatment (28); patients with surgical treatment were reported with significantly lower (*p* = 0.002) recurrence rate than steroid treatment patients (27).

### 3.5 Follow-up duration

**Figure 3:**
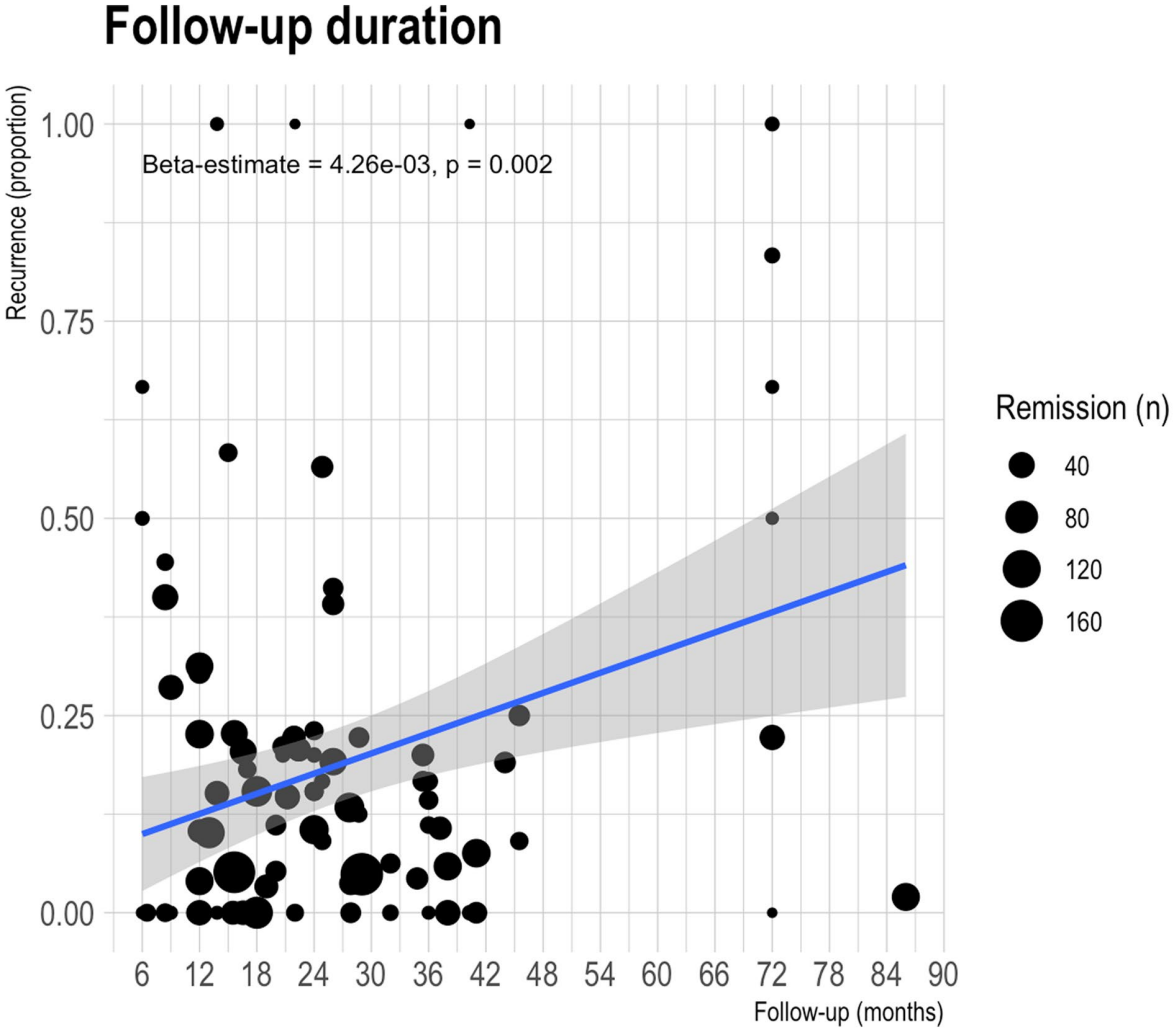
Scatterplot of recurrence proportion against follow-up duration. Each dot represents a treatment modality reported in one of the observational studies included in the meta-analysis. The size of the dot represents the number of patients that achieved remission and were susceptible for developing recurrence. The blue line represents the line of best-fit; the shaded area in grey is the confidence interval.

Follow-up duration was not reported in five studies (31, 57, 62, 66, 79). The remaining 54 studies reported median follow-up duration of 22 months [range 6–86 months] (Table 1). The linear regression models found a significant association between follow-up duration and recurrence rate reported: for every additional month of follow-up, an estimated increase in recurrence rate of 4.26×10^−3^ [1.57×10^−3^-6.95×10^−3^] is observed (*p* = 0.002; Figure 3). This association loses statistical significance when low-powered studies (*n* < 30) are excluded (*p* = 0.273; Supplementary Figure 7).

### 3.6 Quality assessment

Quality assessment with NOS for observational studies and Jadad scoring for randomised studies categorised 35 studies (59.3%) as good, 10 studies (16.9%) as fair, and 14 studies (23.7%) as poor. Among the observational studies, the mean NOS score was 5.86 [interquartile range 5–6]. The randomised studies both scored 3 on the Jadad scale.

### 3.7 Ethical approval

33 studies (55.9%) mentioned ethical approval sought to perform the study: Ahmed, 2016; Alper, 2020; Altunkeser, 2019; Berkesoglu, 2021; Bouton, 2015; Cetin, 2019; Cetinkaya, 2020; Chen, 2021; Chirappapha, 2018; Cornejo-Juarez, 2014; Dalbasi, 2021; Davis, 2019; Erturk, 2021; Farouk, 2017; Fayed, 2019; Joseph, 2014; Kafadar, 2021; Karanlik, 2014; Kiyak, 2014; Maher, 2021; Oak, 2021; Pandey, 2014; Postolova, 2020; Sakurai, 2011; Shin, 2017; Shojaee, 2021; Tan, 2019; Tekgoz, 2020; Toktas, 2021; Wang, 2020; Yabanoglu, 2015; Zhang, 2019; Zhang Xiaohui, 2020. The Çaliş, et al., 2014 and Deng, et al., 2017 studies described no ethical approvals sought, but patients included in the studies had provided written consent (40, 77). The remaining 24 studies (40.7%) did not mention any ethical approval: Akahane, 2013; Akcan, 2014; Aldaqal, 2004; Alrayes, 2019; Altintoprak, 2015; Atak, 2015; Atalay, 2011; Dag, 2021; Elzahaby, 2016; Erhan, 2000; Eser, 2013; Gunduz, 2014; Gurleyik, 2012; Hur, 2013; Kayahan, 2012; Kundaktepe, 2021; Lai, 2005; Liao, 2020; Mawla, 2020; Neel, 2013; Ocal, 2010; Sen Oran, 2013; Skandarajah, 2015; Wang, 2021.

### 3.8 Publication bias

**Figure 4:**
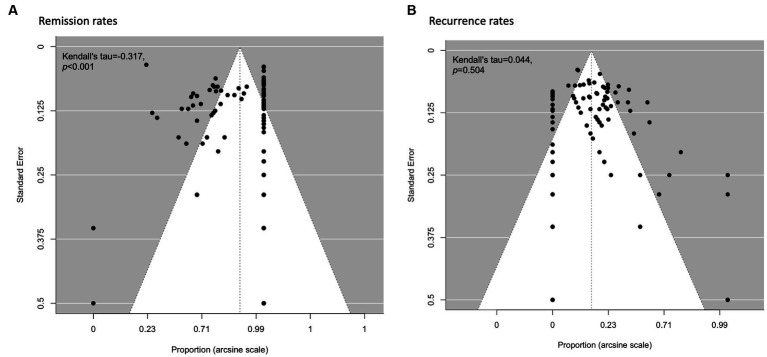
Funnel plots for visualising publication bias for remission and recurrence rates of all treatment types. **(A)** Remission rates. **(B)** Recurrence rates.

Figure 4 shows the funnel plots for assessing publication bias for the estimated pooled remission (Figure 4A) and recurrence (Figure 4B) rates. Begg’s rank correlation test for funnel plot asymmetry indicated statistically significant asymmetry for the reporting on remission rates (*p* < 0.001); this was not statistically significant for recurrence rates (*p* = 0.504).

## 4 Discussion

The studies included in the systematic review highlight substantial variation in study characteristics within published literature on IGM. The rare and elusive nature of the conditions means conducting large-scale, standardised clinical trials to examine treatment efficacy for achieving remission and preventing recurrence is highly challenging (84). As a result, the published articles examining treatment efficacy we have included in this work have diverse approaches in study design, patient populations, and treatment regimens. This diversity in study characteristics implies the heterogeneous nature of the disease, which may require personalised approaches to disease management (85). Recent work has made great efforts in incorporating expert evaluations and evidence-grading of published work for developing clinical practice recommendations for managing IGM patients (86). Our systematic review uniquely contributes statistical evaluations as further evidence-based support for the growing consensus and standardised treatment guidelines being built (86, 87).

Our results have revealed that not only is treatment efficacy widely varied for both remission and recurrence rates reported, heterogeneity within treatment modality is also significant. This further emphasises the complex nature of the disease (88). Despite high remission rates reported in combination therapies with antibiotics, steroids, and surgery, their small sample sizes meant they were underrepresented in the data, so this finding is somewhat limited in its generalisability. Our results also found antibiotics monotherapy had the lowest remission rate, which challenges traditional approaches to treat patients with antibiotics when presented clinically as suspected breast abscesses (6).

Recurrence rates were also subject to variations across different treatment modalities. Observational therapy had the lowest recurrence rate; however, it has been described that patients who feel their medical concerns not properly addressed, will be less likely to return to the same facility when the condition recurs (89). They may have even sought alternative treatments elsewhere at the first instance of IGM, a cofounder for recurrence prevention (90). Patients placed on observation in the observational studies are also more likely to have milder first instances of disease, and this could potentially be a protective factor from recurrence. Surgical interventions and combination therapies also demonstrated relatively low recurrence rates. These findings underscore the need for a personalised approach to IGM treatment, considering individual patient factors, including the severity of the condition and response to initial treatments (85). Additionally, the significant heterogeneity observed in the analysis emphasises the need for further research to better understand which factors influence patients’ response to different treatment approach (91–93).

There is clinical significance to performing future work evaluating treatment effectiveness on the basis of disease severity (94). This will aid clinical decision-making for allocating treatment. Unfortunately, there is no standardised grading metric for the severity of IGM patients (95). Not only do the included articles in our review have a high degree of heterogeneity in severity, both within and across treatment modalities, but severity evaluations are also unlikely to be congruent across different studies. Without standardised measures, severity descriptions would be subjective. There would be insufficient power to perform meaningful statistical analyses within ambiguously established severity measures for treatment efficacy. Furthermore, severity metrics are also unreported in articles included in our review, since severity grading is not standardised clinical practice. With their varied clinical presentations, quantifying or categorising manifestation severity will be challenging. Using an objective scoring tool for disease severity in future studies can potentially aid treatment allocation (95).

The relationship between follow-up duration and recurrence is an important aspect of the study. The results indicated more recurrences diagnosed in studies with longer follow-ups. This association is a crucial consideration for clinicians and highlights the need for extended monitoring of IGM patients to detect and manage any recurrence effectively (96). However, the association loses statistical significance when low-powered studies are excluded, underscoring the need for further research to confirm this relationship and identify potential confounding factors.

While most studies are categorised as good per the respective quality assessment tools, it is important to note that the presence of ethical approval varies among the studies. In some cases, ethical approval was not explicitly mentioned, highlighting the need for standardised reporting of ethical considerations in medical research involving IGM patients. Ethical approval is essential to ensure that research involving human subjects complies with ethical guidelines and respects the rights and well-being of participants (97). In the absence of clear ethical approval, questions may arise about the ethical conduct of the studies (97).

Most studies were conducted in Turkey, followed by China, Egypt, and the United States. This distribution reflects the global interest in IGM research and suggests that IGM is not limited to specific regions (11). Geographical differences in genetics, environmental factors, population density, and healthcare infrastructure can contribute to variations in the presentation and management of IGM (11). The limited standardisation across studies coupled with these geographical differences further questions the comparability of the studies included.

The variability in treatment outcomes and the limited effectiveness of some modalities highlight the need for individualised treatment plans for patients with IGM (85). Moreover, the relatively high recurrence rates emphasise the importance of long-term follow-up and the need for strategies to prevent relapse (91). The call for future prospective studies with larger patient populations is crucial for providing more robust evidence on the efficacy of treatment modalities and long-term outcomes. Randomised prospective studies will improve the reliability and quality of this growing body of evidence (98). Multi-centre collaborations will be crucial for performing robust and rigorous investigations with sufficient patient populations, for the conclusive development of evidence-based approaches to achieve remission and prevent recurrence for IGM patients (99). Clinicians should support and engage in such collaborative prospective research to advance the understanding and management of IGM. Conducting comparative studies directly comparing different treatment modalities can also offer valuable insights into which approaches are the most effective for achieving remission and preventing recurrence. Clinicians should be open to participating in or referring patients to such studies.

Future work must also prioritise obtaining ethical approval and reporting it transparently. This is essential for maintaining the ethical standards of medical research and ensuring patient safety and rights (97). Considering the geographical distribution of IGM studies, future research should also explore how regional factors impact the condition, both in terms of prevalence and treatment outcomes. This can lead to more targeted and region-specific management strategies (11).

There is significant heterogeneity in study characteristics, including the study design, sample sizes, and treatment regimen. Specifically, antibiotic treatment varies in prescription, dosage, and duration; steroid treatments have differing initial and tapering dosages, and duration; surgical treatments differ from case to case, much more so across studies. The definition of disease remission, recurrence monitoring and follow-up duration are important factors that crucially determine the key outcomes reported of remission and recurrence rates; their high heterogeneity in our included studies is problematic. The high heterogeneity limits the generalisability of findings and complicates the comparison of treatment modalities and outcomes. Given the significant variation in study characteristics and treatment approaches, there is a need for standardisation for future work in IGM research, especially in diagnostic criteria, treatment protocols, and reporting guidelines (11).

The severe scarcity of RCTs reduces the clinical robustness of our findings. Most of our findings are based on non-randomised observational studies, which often introduce confounders into the reported data. The limited follow-up information is another limitation: IGM has a recurrent and protracted natural history, and short follow-up periods may not capture long-term recurrence rates accurately (11). The association between follow-up duration and recurrence highlights the need for studies with larger sample sizes that have more extended follow-up durations.

The funnel plot asymmetry implies potential publication bias (100). For the pooled remission rate estimate, smaller studies with less precision may be missing from the left side of the funnel plot; for the recurrence rate estimate, studies with larger effect sizes tend to be higher ranked. While publication bias is a common cause of funnel plot asymmetry, it is important to note that there can be other reasons for asymmetry in funnel plots that are not necessarily indicative of bias (101). Other potential causes relevant to our meta-analysis include heterogeneity in study design, population characteristics, clinical methodologies, and study quality; small study effects; outcome reporting bias; and random variation.

The geographical bias introduced by large portions of the studies included in the analysis recruiting patients from central Eurasia (Turkey, China, Japan, and South Korea) further limits the generalisability of our findings to the global population. Non-traditional treatments are also not evaluated in this study (38, 102). The exclusion of alternative treatments, such as traditional Chinese medication, and minimally invasive procedures, limits the understanding of the full range of treatment options presented to patients (38, 102). More complete clinical data on clinical presentation, disease severity, and treatment response that is not limited to disease remission and recurrence, such as side effects, or partial response, will also greatly improve our understanding of the treatment response in IGM patients.

## 5 Conclusion

The IGM studies included in our systematic review and meta-analysis have highlighted the considerable heterogeneity in treatment outcomes. Combination therapies, especially those incorporating antibiotics, steroids, and surgery, have demonstrated higher remission rates, challenging the traditional use of antibiotic monotherapy. Observation, surgical treatment, and combination therapies showed lower recurrence rates. Our findings also emphasised the critical role of extended follow-up care to detect and manage recurrences effectively. Clinicians and researchers should prioritise randomised prospective studies, multi-centre collaborations and comparative research methodologies to advance evidence-based practices and guide treatment decisions for improved outcomes for patients affected by IGM.

## Data availability statement

The original contributions presented in the study are included in the article/Supplementary material, further inquiries can be directed to the corresponding author.

## Author contributions

SSO: Conceptualization, Formal analysis, Investigation, Methodology, Project administration, Visualization, Writing – original draft, Writing – review & editing. PJH: Formal analysis, Methodology, Supervision, Visualization, Writing – review & editing. JJKL: Methodology, Writing – review & editing. QTT: Methodology, Writing – review & editing. SSNG: Methodology, Writing – review & editing. JL: Conceptualization, Methodology, Project administration, Resources, Supervision, Writing – review & editing. MH: Conceptualization, Funding acquisition, Supervision, Writing – review & editing.
