# Supplementary material for: A meta-analysis of idiopathic granulomatous mastitis treatments for remission and recurrence prevention
Source: Front Med (Lausanne). 2024 May 30;11:1346790. doi: 10.3389/fmed.2024.1346790 (PMC11170159; doi:10.3389/fmed.2024.1346790)
Supplement: Supplementary file 1 [file Data_Sheet_1.DOCX]

Supplementary Material

# Supplementary Tables

Supplementary table 1. Overview of treatment modalities, and their pooled estimates for remission and recurrence rates. n: sample size of patients, reported in each study; CI: confidence interval.

|  |  |  |  |  | Remission | | Recurrence | |
| --- | --- | --- | --- | --- | --- | --- | --- | --- |
| Treatment strategy | **Number of studies** | **n, median [range]** | **Remission, median [range]** | **Recurrence, median [range]** | **Pooled effect estimate, proportion [95%CI]** | **Heterogeneity, %** | **Pooled effect estimate, proportion [95%CI]** | **Heterogeneity, %** |
| Observation | 11 | 7 [1-112] | 4 [0-112] | 0 [0-119] | 0.91 [0.73-0.99] | 87.0 | 0.03 [0.00-0.10] | 56.2 |
| Antibiotics | 9 | 11 [2-30] | 9 [0-30] | 0 [0-5] | 0.72 [0.37-0.96] | 92.6 | 0.14 [0.00-0.41] | 88.1 |
| Steroids | 26 | 19 [2-200] | 18 [2-44] | 2 [0-10] | 0.93 [0.85-0.98] | 89.9 | 0.13 [0.08-0.20] | 74.9 |
| *Peroral Steroids* | 20 | 13 [2-200] | 12 [2-44] | 1.5 [0-10] | 0.92 [0.81-0.98] | 91.1 | 0.17 [0.10-0.25] | 66.1 |
| *Injected Steroids* | 2 | 33 [28-38] | 31 [26-36] | 0 [0-0] | NA | NA | NA | NA |
| *Topical Steroids* | 3 | 28 [11-41] | 28 [11-34] | 3 [2-5] | 0.98 [0.81-1.00] | 83.9 | 0.14 [0.07-0.22] | 0 |
| *Peroral and Topical Steroids* | 1 | 33 | 27 | 6 | NA | NA | NA | NA |
| Methotrexate | 3 | 19 [17-60] | 12 [10-60] | 2 [0-8] | 0.82 [0.40-1.00] | 93.9 | 0.08 [0.00-0.24] | 64.3 |
| Surgery | 37 | 15 [1-164] | 14 [1-164] | 1 [0-15] | 0.99 [0.97-1.00] | 73.6 | 0.15 [0.09-0.22] | 64.6 |
| *Surgery (n≥30)* | 8 | 40.5 [30-164] | 40.5 [19-164] | 3 [0-15] | 0.99 [0.94-1.00] | 90.0 | 0.08 [0.02-0.15] | 83.9 |
| *Biopsy and aspiration only* | 5 | 9 [1-29] | 9 [1-21] | 1 [1-12] | 0.98 [0.83-1.00] | 72.8 | 0.41 [0.08-0.80] | 86.5 |
| *Excisional* | 31 | 15 [1-68] | 14 [1-68] | 1 [0-15] | 0.99 [0.96-1.00] | 69.8 | 0.13 [0.08-0.19] | 64.8 |
| *Aspiration and Excision* | 1 | 164 | 164 | 8 | NA | NA | NA | NA |
|  |  |  |  |  |  |  |  |  |
| *Combination Therapy* | |  |  |  |  |  |  |  |
| Antibiotics, and steroids | 8 | 7 [3-41] | 7 [2-40] | 0 [0-16] | 0.94 [0.81-1.00] | 68.8 | 0.08 [0.00-0.30] | 84.1 |
| Antibiotics, and methotrexate | 1 | 1 | 1 | 1 | NA | NA | NA | NA |
| Antibiotics, and surgery | 5 | 4 [1-29] | 4 [1-29] | 1 [0-4] | 0.93 [0.53-1.00] | 86.6 | 0.54 [0.02-1.00] | 89.6 |
| Steroids, and methotrexate | 2 | 49 [41-57] | 45 [33-57] | 5.5 [5-6] | NA | NA | NA | NA |
| Steroids, and surgery | 10 | 29 [1-156] | 29 [1-156] | 1 [0-12] | 0.99 [0.94-1.00] | 82.4 | 0.05 [0.01-0.12] | 78.3 |
| Methotrexate, and surgery | 1 | 2 | 2 | 0 | NA | NA | NA | NA |
| Antibiotics, steroids, and methotrexate | 1 | 1 | 1 | 0 | NA | NA | NA | NA |
| Antibiotics, steroids, and surgery | 3 | 1 [1-3] | 1 [1-3] | 1 [0-2] | 1.00 [0.82-1.00] | 0 | 0.57 [0.00-1.00] | 63.6 |
| Antibiotics, methotrexate, and surgery | 1 | 1 | 1 | 0 | NA | NA | NA | NA |
| Steroids, methotrexate, and surgery | 1 | 5 | 5 | 1 | NA | NA | NA | NA |
| Antibiotics, steroids, methotrexate, and surgery | 1 | 2 | 2 | 0 | NA | NA | NA | NA |

# Supplementary figure captions

Supplementary figure 1. Forest plots for remission and recurrence rates for observation. (A) Estimated pooled remission rate. (B) Estimated pooled recurrence rate.

Supplementary figure 2. Forest plots for remission and recurrence rates for antibiotic monotherapy. (A) Estimated pooled remission rate. (B) Estimated pooled recurrence rate.

Supplementary figure 3. Forest plots for remission and recurrence rates for variations of steroid treatment. (A) Estimated pooled remission rate for treatment modalities with steroid treatment only: peroral steroids, injected steroids, topical steroids, and peroral and topical steroids. (B) Estimated pooled recurrence rate for treatment modalities with steroid treatment only: peroral steroids, injected steroids, topical steroids, and peroral and topical steroids. (C) Estimated pooled remission rate for peroral steroid monotherapy. (D) Estimated pooled recurrence rate for peroral steroid monotherapy. (E) Estimated pooled remission rate for topical steroid monotherapy. (F) Estimated pooled recurrence rate for topical steroid monotherapy.

Supplementary figure 4. Forest plots for remission and recurrence rates for methotrexate monotherapy. (A) Estimated pooled remission rate. (B) Estimated pooled recurrence rate.

Supplementary figure 5. Forest plots for remission and recurrence rates for variations of surgical treatment. (A) Estimated pooled remission rate for treatment modalities with surgical treatment only: biopsy and aspiration only, excisional, and aspiration and excision. (B) Estimated pooled recurrence rate for treatment modalities with steroid treatment only: biopsy and aspiration only, excisional, and aspiration and excision. (C) Same as (A), but only pooling studies with higher statistical power (n≥30). (D) Same as (B), but only pooling studies with higher statistical power (remission≥30). (E) Estimated pooled remission rate for biopsy and aspiration procedure only. (F) Estimated pooled recurrence rate for biopsy and aspiration procedure only. (G) Estimated pooled remission rate for excisional procedure only. (H) Estimated pooled recurrence rate for excisional procedure only.

Supplementary figure 6. Forest plots for remission and recurrence rates for combination therapies. (A) Estimated pooled remission rate for antibiotics and steroids combination therapy. (B) Estimated pooled recurrence rate for antibiotics and steroids combination therapy. (C) Estimated pooled remission rate for antibiotics and surgery combination therapy. (D) Estimated pooled recurrence rate for antibiotics and surgery combination therapy. (E) Estimated pooled remission rate for steroids and surgery combination therapy. (F) Estimated pooled recurrence rate for steroids and surgery combination therapy. (G) Estimated pooled remission rate for antibiotics, steroids, and surgery combination therapy. (H) Estimated pooled recurrence rate for antibiotics, steroids, and surgery combination therapy.

Supplementary figure 7. Scatterplot of recurrence proportion against follow-up duration, for treatment modalities reported with n≥30. Each dot represents a treatment modality reported in one of the observational studies included in the meta-analysis. The size of the dot represents the number of patients that achieved remission, and were susceptible for developing recurrence. The blue line represents the line of best-fit; the shaded area in grey is the confidence interval.

# Appendix A

PubMed Search (Date: 19 January 2022)

| Disease | "granulomatous mastitis"[MeSH Terms] OR (("granuloma"[MeSH Terms] OR "granulomatous disease, chronic"[MeSH Terms] OR "granulomatous disease, chronic"[MeSH Terms] OR "granulomatous disease, chronic"[MeSH Terms] OR "granulomatous disease, chronic"[MeSH Terms]) AND ("mastitis"[MeSH Terms] OR ("breast diseases"[MeSH Terms] OR "breast diseases"[MeSH Terms]) OR ("breast"[MeSH Terms] AND ("inflammation"[MeSH Terms] OR "infections"[MeSH Terms])))) OR ("idiopathic granulomatous mastitis"[Title/Abstract] OR "IGM"[Title/Abstract] OR ("granuloma*"[Title/Abstract] AND ("mastitis*"[Title/Abstract] OR ("breast disease*"[Title/Abstract] OR "breast inflam*"[Title/Abstract] OR "breast infect*"[Title/Abstract])))) |
| --- | --- |
| Treatment | "therapeutics"[MeSH Terms] OR "general surgery"[MeSH Terms] OR "mastectomy, simple"[MeSH Terms] OR "mastectomy"[MeSH Terms] OR "steroids"[MeSH Terms] OR "anti bacterial agents"[MeSH Terms] OR "anti bacterial agents"[MeSH Terms] OR "anti bacterial agents"[MeSH Terms] OR "methotrexate"[MeSH Terms] OR "disease management"[MeSH Terms] OR "disease management"[MeSH Terms] OR "treat*"[Title/Abstract] OR "therap*"[Title/Abstract] OR "surger*"[Title/Abstract] OR "mastectom*"[Title/Abstract] OR "lumpectom*"[Title/Abstract] OR "excis*"[Title/Abstract] OR "incis*"[Title/Abstract] OR "drain*"[Title/Abstract] OR "steroid*"[Title/Abstract] OR "corticosteroid*"[Title/Abstract] OR "prednisolone"[Title/Abstract] OR "antibiotic*"[Title/Abstract] OR "augmentin"[Title/Abstract] OR "amoxicillin"[Title/Abstract] OR "amoxicilin"[Title/Abstract] OR "metronidazole"[Title/Abstract] OR "flagyl"[Title/Abstract] OR "methotrexate"[Title/Abstract] |
| Outcome | "remission induction"[MeSH Terms] OR "remission induction"[MeSH Terms] OR "remission induction"[MeSH Terms] OR "remission induction"[MeSH Terms] OR "remission, spontaneous"[MeSH Terms] OR "remission, spontaneous"[MeSH Terms] OR "recurrence"[MeSH Terms] OR "prognosis"[MeSH Terms] OR "remiss*"[Title/Abstract] OR "recur*"[Title/Abstract] OR "prognos*"[Title/Abstract] |
| Combined | ("granulomatous mastitis"[MeSH Terms] OR (("granuloma"[MeSH Terms] OR "granulomatous disease, chronic"[MeSH Terms] OR "granulomatous disease, chronic"[MeSH Terms] OR "granulomatous disease, chronic"[MeSH Terms] OR "granulomatous disease, chronic"[MeSH Terms]) AND ("mastitis"[MeSH Terms] OR ("breast diseases"[MeSH Terms] OR "breast diseases"[MeSH Terms]) OR ("breast"[MeSH Terms] AND ("inflammation"[MeSH Terms] OR "infections"[MeSH Terms])))) OR ("idiopathic granulomatous mastitis"[Title/Abstract] OR "IGM"[Title/Abstract] OR ("granuloma*"[Title/Abstract] AND ("mastitis*"[Title/Abstract] OR ("breast disease*"[Title/Abstract] OR "breast inflam*"[Title/Abstract] OR "breast infect*"[Title/Abstract]))))) AND ("therapeutics"[MeSH Terms] OR "general surgery"[MeSH Terms] OR ("mastectomy, simple"[MeSH Terms] OR "mastectomy"[MeSH Terms]) OR "steroids"[MeSH Terms] OR "anti bacterial agents"[MeSH Terms] OR "anti bacterial agents"[MeSH Terms] OR "anti bacterial agents"[MeSH Terms] OR "methotrexate"[MeSH Terms] OR "disease management"[MeSH Terms] OR "disease management"[MeSH Terms] OR ("treat*"[Title/Abstract] OR "therap*"[Title/Abstract] OR "surger*"[Title/Abstract] OR "mastectom*"[Title/Abstract] OR "lumpectom*"[Title/Abstract] OR "excis*"[Title/Abstract] OR "incis*"[Title/Abstract] OR "drain*"[Title/Abstract] OR "steroid*"[Title/Abstract] OR "corticosteroid*"[Title/Abstract] OR "prednisolone"[Title/Abstract] OR "antibiotic*"[Title/Abstract] OR "augmentin"[Title/Abstract] OR "amoxicillin"[Title/Abstract] OR "amoxicilin"[Title/Abstract] OR "metronidazole"[Title/Abstract] OR "flagyl"[Title/Abstract] OR "methotrexate"[Title/Abstract])) AND ("remission induction"[MeSH Terms] OR "remission induction"[MeSH Terms] OR "remission induction"[MeSH Terms] OR "remission induction"[MeSH Terms] OR "remission, spontaneous"[MeSH Terms] OR "remission, spontaneous"[MeSH Terms] OR "recurrence"[MeSH Terms] OR "prognosis"[MeSH Terms] OR ("remiss*"[Title/Abstract] OR "recur*"[Title/Abstract] OR "prognos*"[Title/Abstract])) |

Medline (OVID) Search (Date: 19 Janaury 2022)

| Disease | granulomatous mastitis/ or ((granuloma/ or granulomatous disease, chronic/ or granulomatous diseases, chronic/ or chronic granulomatous disease/ or chronic granulomatous diseases/) and (mastitis/ or (breast disease/ or breast diseases/) or (breast/ and (inflammation/ or infections/)))) or ((idiopathic granulomatous mastitis or IGM or (granuloma* and (mastitis* or (breast disease* or breast inflam* or breast infect*)))).ti. or (idiopathic granulomatous mastitis or IGM or (granuloma* and (mastitis* or (breast disease* or breast inflam* or breast infect*)))).ab.) |
| --- | --- |
| Treatment | therapeutics/ or general surgery/ or mastectomy/ or steroids/ or antibiotics/ or agents, antibacterial/ or antibacterial agents/ or methotrexate/ or disease management/ or disease managements/ or ((treat* or therap* or surger* or mastectom* or lumpectom* or excis* or incis* or drain* or steroid* or corticosteroid* or prednisolone or antibiotic* or augmentin or amoxicillin or amoxicilin or metronidazole or flagyl or methotrexate).ti. or (treat* or therap* or surger* or mastectom* or lumpectom* or excis* or incis* or drain* or steroid* or corticosteroid* or prednisolone or antibiotic* or augmentin or amoxicillin or amoxicilin or metronidazole or flagyl or methotrexate).ab.) |
| Outcome | induction of remission/ or induction, remission/ or inductions, remission/ or remission induction/ or spontaneous remission/ or spontaneous remissions/ or recurrence/ or prognosis/ or ((remiss* or recur* or prognos*).ti. or (remiss* or recur* or prognos*).ab.) |
| Combined | (granulomatous mastitis/ or ((granuloma/ or granulomatous disease, chronic/ or granulomatous diseases, chronic/ or chronic granulomatous disease/ or chronic granulomatous diseases/) and (mastitis/ or (breast disease/ or breast diseases/) or (breast/ and (inflammation/ or infections/)))) or ((idiopathic granulomatous mastitis or IGM or (granuloma* and (mastitis* or (breast disease* or breast inflam* or breast infect*)))).ti. or (idiopathic granulomatous mastitis or IGM or (granuloma* and (mastitis* or (breast disease* or breast inflam* or breast infect*)))).ab.)) and (therapeutics/ or general surgery/ or mastectomy/ or steroids/ or antibiotics/ or agents, antibacterial/ or antibacterial agents/ or methotrexate/ or disease management/ or disease managements/ or ((treat* or therap* or surger* or mastectom* or lumpectom* or excis* or incis* or drain* or steroid* or corticosteroid* or prednisolone or antibiotic* or augmentin or amoxicillin or amoxicilin or metronidazole or flagyl or methotrexate).ti. or (treat* or therap* or surger* or mastectom* or lumpectom* or excis* or incis* or drain* or steroid* or corticosteroid* or prednisolone or antibiotic* or augmentin or amoxicillin or amoxicilin or metronidazole or flagyl or methotrexate).ab.)) and (induction of remission/ or induction, remission/ or inductions, remission/ or remission induction/ or spontaneous remission/ or spontaneous remissions/ or recurrence/ or prognosis/ or ((remiss* or recur* or prognos*).ti. or (remiss* or recur* or prognos*).ab.)) |

Embase (Elsevier) Search (Date: 22 January 2022)

| Disease | ('granulomatous mastitis'/exp/mj OR (('granuloma'/exp/mj OR 'granulomatous disease, chronic'/exp/mj OR 'granulomatous diseases, chronic' OR 'chronic granulomatous disease'/exp/mj OR 'chronic granulomatous diseases') AND ('mastitis'/exp/mj OR 'breast disease'/exp/mj OR 'breast diseases'/exp/mj OR ('breast'/exp/mj AND ('inflammation'/exp/mj OR 'infections'/exp/mj)))) OR 'idiopathic granulomatous mastitis':ti OR 'igm':ti OR ('granuloma*':ti AND ('mastitis*':ti OR 'breast disease*':ti OR 'breast inflam*':ti OR 'breast infect*':ti)) OR 'idiopathic granulomatous mastitis':ab OR 'igm':ab OR ('granuloma*':ab AND ('mastitis*':ab OR 'breast disease*':ab OR 'breast inflam*':ab OR 'breast infect*':ab))) AND [01-01-1000]/sd NOT [20-01-2022]/sd |
| --- | --- |
| Treatment | ('therapeutics'/exp/mj OR 'general surgery'/exp/mj OR 'mastectomy, simple'/exp/mj OR 'mastectomy'/exp/mj OR 'steroids'/exp/mj OR 'anti bacterial agents'/exp/mj OR 'methotrexate'/exp/mj OR 'disease management'/exp/mj OR 'treat*':ti OR 'therap*':ti OR 'surger*':ti OR 'mastectom*':ti OR 'lumpectom*':ti OR 'excis*':ti OR 'incis*':ti OR 'drain*':ti OR 'steroid*':ti OR 'corticosteroid*':ti OR 'prednisolone':ti OR 'antibiotic*':ti OR 'augmentin':ti OR 'amoxicillin':ti OR 'amoxicilin':ti OR 'metronidazole':ti OR 'flagyl':ti OR 'methotrexate':ti OR 'treat*':ab OR 'therap*':ab OR 'surger*':ab OR 'mastectom*':ab OR 'lumpectom*':ab OR 'excis*':ab OR 'incis*':ab OR 'drain*':ab OR 'steroid*':ab OR 'corticosteroid*':ab OR 'prednisolone':ab OR 'antibiotic*':ab OR 'augmentin':ab OR 'amoxicillin':ab OR 'amoxicilin':ab OR 'metronidazole':ab OR 'flagyl':ab OR 'methotrexate':ab) AND [01-01-1000]/sd NOT [20-01-2022]/sd |
| Outcome | ('remission induction'/exp/mj OR 'remission, spontaneous'/exp/mj OR 'recurrence'/exp/mj OR 'prognosis'/exp/mj OR 'remiss*':ti OR 'recur*':ti OR 'prognos*':ti OR 'remiss*':ab OR 'recur*':ab OR 'prognos*':ab) AND [01-01-1000]/sd NOT [20-01-2022]/sd |
| Combined | (('granulomatous mastitis'/exp/mj OR (('granuloma'/exp/mj OR 'granulomatous disease, chronic'/exp/mj OR 'granulomatous diseases, chronic' OR 'chronic granulomatous disease'/exp/mj OR 'chronic granulomatous diseases') AND ('mastitis'/exp/mj OR 'breast disease'/exp/mj OR 'breast diseases'/exp/mj OR ('breast'/exp/mj AND ('inflammation'/exp/mj OR 'infections'/exp/mj)))) OR 'idiopathic granulomatous mastitis':ti OR 'igm':ti OR ('granuloma*':ti AND ('mastitis*':ti OR 'breast disease*':ti OR 'breast inflam*':ti OR 'breast infect*':ti)) OR 'idiopathic granulomatous mastitis':ab OR 'igm':ab OR ('granuloma*':ab AND ('mastitis*':ab OR 'breast disease*':ab OR 'breast inflam*':ab OR 'breast infect*':ab))) and ('therapeutics'/exp/mj OR 'general surgery'/exp/mj OR 'mastectomy, simple'/exp/mj OR 'mastectomy'/exp/mj OR 'steroids'/exp/mj OR 'anti bacterial agents'/exp/mj OR 'methotrexate'/exp/mj OR 'disease management'/exp/mj OR 'treat*':ti OR 'therap*':ti OR 'surger*':ti OR 'mastectom*':ti OR 'lumpectom*':ti OR 'excis*':ti OR 'incis*':ti OR 'drain*':ti OR 'steroid*':ti OR 'corticosteroid*':ti OR 'prednisolone':ti OR 'antibiotic*':ti OR 'augmentin':ti OR 'amoxicillin':ti OR 'amoxicilin':ti OR 'metronidazole':ti OR 'flagyl':ti OR 'methotrexate':ti OR 'treat*':ab OR 'therap*':ab OR 'surger*':ab OR 'mastectom*':ab OR 'lumpectom*':ab OR 'excis*':ab OR 'incis*':ab OR 'drain*':ab OR 'steroid*':ab OR 'corticosteroid*':ab OR 'prednisolone':ab OR 'antibiotic*':ab OR 'augmentin':ab OR 'amoxicillin':ab OR 'amoxicilin':ab OR 'metronidazole':ab OR 'flagyl':ab OR 'methotrexate':ab) and ('remission induction'/exp/mj OR 'remission, spontaneous'/exp/mj OR 'recurrence'/exp/mj OR 'prognosis'/exp/mj OR 'remiss*':ti OR 'recur*':ti OR 'prognos*':ti OR 'remiss*':ab OR 'recur*':ab OR 'prognos*':ab)) AND [01-01-1000]/sd NOT [20-01-2022]/sd |

Cochrane Library Search (Date: 19 January 2022)

| Disease | ((granulomatous mastitis) OR (((granuloma) OR (granulomatous disease, chronic) OR (granulomatous disease, chronic) OR (granulomatous disease, chronic) OR (granulomatous disease, chronic)) AND ((mastitis) OR ((breast diseases) OR (breast diseases)) OR ((breast) AND ((inflammation) OR (infections)))))):kw OR (((idiopathic granulomatous mastitis) OR (IGM) OR ((granuloma*) AND ((mastitis*) OR ((breast disease*) OR (breast inflam*) OR (breast infect*)))))):ti OR (((idiopathic granulomatous mastitis) OR (IGM) OR ((granuloma*) AND ((mastitis*) OR ((breast disease*) OR (breast inflam*) OR (breast infect*)))))):ab |
| --- | --- |
| Treatment | ((therapeutics) OR (general surgery) OR (mastectomy) OR (steroids) OR (antibiotics) OR (agents, antibacterial) OR (antibacterial agents) OR (methotrexate) OR (disease management) OR (disease managements)):kw OR ((treat*) OR (therap*) OR (surger*) OR (mastectom*) OR (lumpectom*) OR (excis*) OR (incis*) OR (drain*) OR (steroid*) OR (corticosteroid*) OR (prednisolone) OR (antibiotic*) OR (augmentin) OR (amoxicillin) OR (amoxicilin) OR (metronidazole) OR (flagyl) OR (methotrexate)):ti OR ((treat*) OR (therap*) OR (surger*) OR (mastectom*) OR (lumpectom*) OR (excis*) OR (incis*) OR (drain*) OR (steroid*) OR (corticosteroid*) OR (prednisolone) OR (antibiotic*) OR (augmentin) OR (amoxicillin) OR (amoxicilin) OR (metronidazole) OR (flagyl) OR (methotrexate)):ab |
| Outcome | ((induction of remission) OR (induction, remission) OR (inductions, remission) OR (remission induction) OR (spontaneous remission) OR (spontaneous remissions) OR (recurrence) OR (prognosis)):kw OR ((remiss*) OR (recur*) OR (prognos*)):ti OR ((remiss*) OR (recur*) OR (prognos*)):ab |
| Combined | (((granulomatous mastitis) OR (((granuloma) OR (granulomatous disease, chronic) OR (granulomatous disease, chronic) OR (granulomatous disease, chronic) OR (granulomatous disease, chronic)) AND ((mastitis) OR ((breast diseases) OR (breast diseases)) OR ((breast) AND ((inflammation) OR (infections)))))):kw OR (((idiopathic granulomatous mastitis) OR (IGM) OR ((granuloma*) AND ((mastitis*) OR ((breast disease*) OR (breast inflam*) OR (breast infect*)))))):ti OR (((idiopathic granulomatous mastitis) OR (IGM) OR ((granuloma*) AND ((mastitis*) OR ((breast disease*) OR (breast inflam*) OR (breast infect*)))))):ab) AND (((therapeutics) OR (general surgery) OR (mastectomy) OR (steroids) OR (antibiotics) OR (agents, antibacterial) OR (antibacterial agents) OR (methotrexate) OR (disease management) OR (disease managements)):kw OR ((treat*) OR (therap*) OR (surger*) OR (mastectom*) OR (lumpectom*) OR (excis*) OR (incis*) OR (drain*) OR (steroid*) OR (corticosteroid*) OR (prednisolone) OR (antibiotic*) OR (augmentin) OR (amoxicillin) OR (amoxicilin) OR (metronidazole) OR (flagyl) OR (methotrexate)):ti OR ((treat*) OR (therap*) OR (surger*) OR (mastectom*) OR (lumpectom*) OR (excis*) OR (incis*) OR (drain*) OR (steroid*) OR (corticosteroid*) OR (prednisolone) OR (antibiotic*) OR (augmentin) OR (amoxicillin) OR (amoxicilin) OR (metronidazole) OR (flagyl) OR (methotrexate)):ab) AND (((induction of remission) OR (induction, remission) OR (inductions, remission) OR (remission induction) OR (spontaneous remission) OR (spontaneous remissions) OR (recurrence) OR (prognosis)):kw OR ((remiss*) OR (recur*) OR (prognos*)):ti OR ((remiss*) OR (recur*) OR (prognos*)):ab) |

Web of Science Search (Date: 22 January 2022)

| Disease | (AK=(granulomatous mastitis or ((granuloma or granulomatous disease, chronic or granulomatous diseases, chronic or chronic granulomatous disease or chronic granulomatous diseases) and (mastitis or (breast disease or breast diseases) or (breast and (inflammation or infections))))) OR (TI=(idiopathic granulomatous mastitis or IGM or (granuloma* and (mastitis* or (breast disease* or breast inflam* or breast infect*)))) OR AB=(idiopathic granulomatous mastitis or IGM or (granuloma* and (mastitis* or (breast disease* or breast inflam* or breast infect*)))))) AND DOP=(1000-01-01/2022-01-19) |
| --- | --- |
| Treatment | (AK=(therapeutics or general surgery or mastectomy or steroids or antibiotics or agents, antibacterial or antibacterial agents or methotrexate or disease management or disease managements) OR (TI=(treat* or therap* or surger* or mastectom* or lumpectom* or excis* or incis* or drain* or steroid* or corticosteroid* or prednisolone or antibiotic* or augmentin or amoxicillin or amoxicilin or metronidazole or flagyl or methotrexate) OR AB=(treat* or therap* or surger* or mastectom* or lumpectom* or excis* or incis* or drain* or steroid* or corticosteroid* or prednisolone or antibiotic* or augmentin or amoxicillin or amoxicilin or metronidazole or flagyl or methotrexate))) AND DOP=(1000-01-01/2022-01-19) |
| Outcome | (AK=(induction of remission or induction, remission or inductions, remission or remission induction or spontaneous remission or spontaneous remissions or recurrence or prognosis) OR (TI=(remiss* or recur* or prognos*) OR AB=(remiss* or recur* or prognos*))) AND DOP=(1000-01-01/2022-01-19) |
| Combined | (AK=(granulomatous mastitis or ((granuloma or granulomatous disease, chronic or granulomatous diseases, chronic or chronic granulomatous disease or chronic granulomatous diseases) and (mastitis or (breast disease or breast diseases) or (breast and (inflammation or infections))))) OR (TI=(idiopathic granulomatous mastitis or IGM or (granuloma* and (mastitis* or (breast disease* or breast inflam* or breast infect*)))) OR AB=(idiopathic granulomatous mastitis or IGM or (granuloma* and (mastitis* or (breast disease* or breast inflam* or breast infect*)))))) AND (AK=(therapeutics or general surgery or mastectomy or steroids or antibiotics or agents, antibacterial or antibacterial agents or methotrexate or disease management or disease managements) OR (TI=(treat* or therap* or surger* or mastectom* or lumpectom* or excis* or incis* or drain* or steroid* or corticosteroid* or prednisolone or antibiotic* or augmentin or amoxicillin or amoxicilin or metronidazole or flagyl or methotrexate) OR AB=(treat* or therap* or surger* or mastectom* or lumpectom* or excis* or incis* or drain* or steroid* or corticosteroid* or prednisolone or antibiotic* or augmentin or amoxicillin or amoxicilin or metronidazole or flagyl or methotrexate))) AND (AK=(induction of remission or induction, remission or inductions, remission or remission induction or spontaneous remission or spontaneous remissions or recurrence or prognosis) OR (TI=(remiss* or recur* or prognos*) OR AB=(remiss* or recur* or prognos*))) AND DOP=(1000-01-01/2022-01-19) |

Google Scholar Search (Date: 19 Janaury 2022)

| Disease | allintitle: "idiopathic granulomatous mastitis" OR "IGM" OR ((granuloma OR granulomatous) AND (mastitis OR (breast AND (disease OR diseases OR inflammation OR inflammatory OR infection OR infections)))) |
| --- | --- |
| Treatment | allintitle: treatment OR treat OR treats OR therapies OR therapy OR surgery OR surgeries OR mastectomy OR mastectomies OR lumpectomy OR lumpectomies OR excision OR excisions OR incision OR incisions OR drain OR drainage OR drainages OR steroid OR steroids OR corticosteroid OR corticosteroids OR prednisolone OR antibiotic OR antibiotics OR augmentin OR amoxicillin OR amoxicilin OR metronidazole OR flagyl OR methotrexate |
| Outcome | allintitle: remission OR remissions OR recur OR recurrence OR recurs OR recurrences OR prognosis OR prognosticate OR prognoses OR prognosticates |
| Combined | allintitle: ("idiopathic granulomatous mastitis" OR "IGM" OR ((granuloma OR granulomatous) AND (mastitis OR (breast (disease OR diseases OR inflammation OR inflammatory OR infection OR infections))))) AND (treatment OR treat OR treats OR therapies OR therapy OR surgery OR surgeries OR mastectomy OR mastectomies OR lumpectomy OR lumpectomies OR excision OR excisions OR incision OR incisions OR drain OR drainage OR drainages OR steroid OR steroids OR corticosteroid OR corticosteroids OR prednisolone OR antibiotic OR antibiotics OR augmentin OR amoxicillin OR amoxicilin OR metronidazole OR flagyl OR methotrexate) AND (remission OR remissions OR recur OR recurrence OR recurs OR recurrences OR prognosis OR prognosticate OR prognoses OR prognosticates) |

Scopus Search (Date: 19 January 2022)

| Disease | KEY(((granulomatous mastitis) OR (((granuloma) OR (granulomatous disease, chronic) OR (granulomatous disease, chronic) OR (granulomatous disease, chronic) OR (granulomatous disease, chronic)) AND ((mastitis) OR ((breast diseases) OR (breast diseases)) OR ((breast) AND ((inflammation) OR (infections))))))) OR TITLE-ABS(((idiopathic granulomatous mastitis) OR (IGM) OR ((granuloma*) AND ((mastitis*) OR ((breast disease*) OR (breast inflam*) OR (breast infect*)))))) |
| --- | --- |
| Treatment | KEY((therapeutics) OR (general surgery) OR (mastectomy) OR (steroids) OR (antibiotics) OR (agents, antibacterial) OR (antibacterial agents) OR (methotrexate) OR (disease management) OR (disease managements)) OR TITLE-ABS((treat*) OR (therap*) OR (surger*) OR (mastectom*) OR (lumpectom*) OR (excis*) OR (incis*) OR (drain*) OR (steroid*) OR (corticosteroid*) OR (prednisolone) OR (antibiotic*) OR (augmentin) OR (amoxicillin) OR (amoxicilin) OR (metronidazole) OR (flagyl) OR (methotrexate)) |
| Outcome | KEY((induction of remission) OR (induction, remission) OR (inductions, remission) OR (remission induction) OR (spontaneous remission) OR (spontaneous remissions) OR (recurrence) OR (prognosis)) OR TITLE-ABS((remiss*) OR (recur*) OR (prognos*)) |
| Combined | (KEY(((granulomatous mastitis) OR (((granuloma) OR (granulomatous disease, chronic) OR (granulomatous disease, chronic) OR (granulomatous disease, chronic) OR (granulomatous disease, chronic)) AND ((mastitis) OR ((breast diseases) OR (breast diseases)) OR ((breast) AND ((inflammation) OR (infections))))))) OR TITLE-ABS(((idiopathic granulomatous mastitis) OR (IGM) OR ((granuloma*) AND ((mastitis*) OR ((breast disease*) OR (breast inflam*) OR (breast infect*))))))) AND (KEY((therapeutics) OR (general surgery) OR (mastectomy) OR (steroids) OR (antibiotics) OR (agents, antibacterial) OR (antibacterial agents) OR (methotrexate) OR (disease management) OR (disease managements)) OR TITLE-ABS((treat*) OR (therap*) OR (surger*) OR (mastectom*) OR (lumpectom*) OR (excis*) OR (incis*) OR (drain*) OR (steroid*) OR (corticosteroid*) OR (prednisolone) OR (antibiotic*) OR (augmentin) OR (amoxicillin) OR (amoxicilin) OR (metronidazole) OR (flagyl) OR (methotrexate))) AND (KEY((induction of remission) OR (induction, remission) OR (inductions, remission) OR (remission induction) OR (spontaneous remission) OR (spontaneous remissions) OR (recurrence) OR (prognosis)) OR TITLE-ABS((remiss*) OR (recur*) OR (prognos*))) |
