# Supplementary figures and images for: A meta-analysis of idiopathic granulomatous mastitis treatments for remission and recurrence prevention
Source: Front Med (Lausanne). 2024 May 30;11:1346790. doi: 10.3389/fmed.2024.1346790 (PMC11170159; doi:10.3389/fmed.2024.1346790)

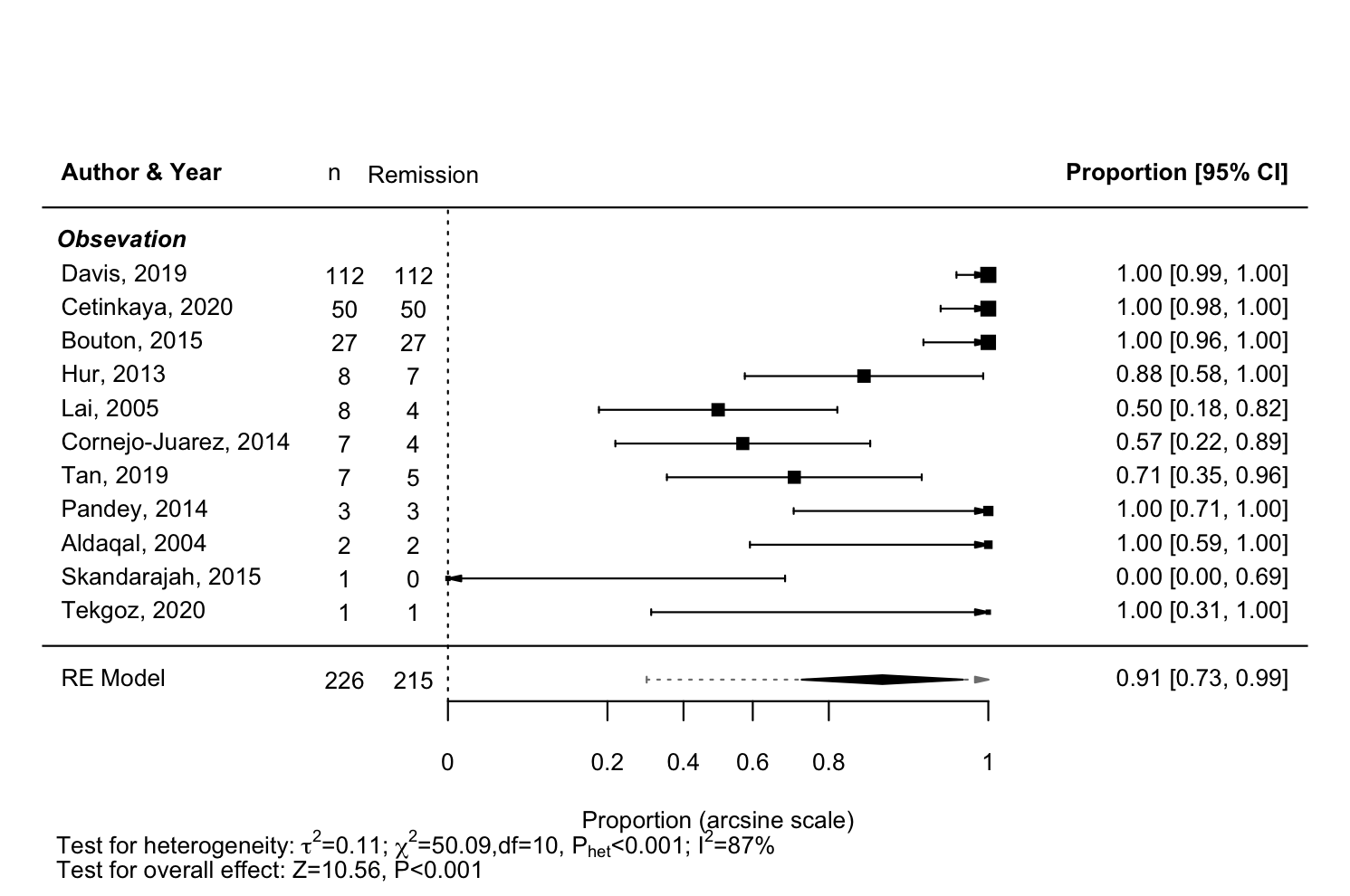

Supplement: Supplementary file 2 [file Presentation_1.ZIP › Supplementary Figure 1a.jpg]

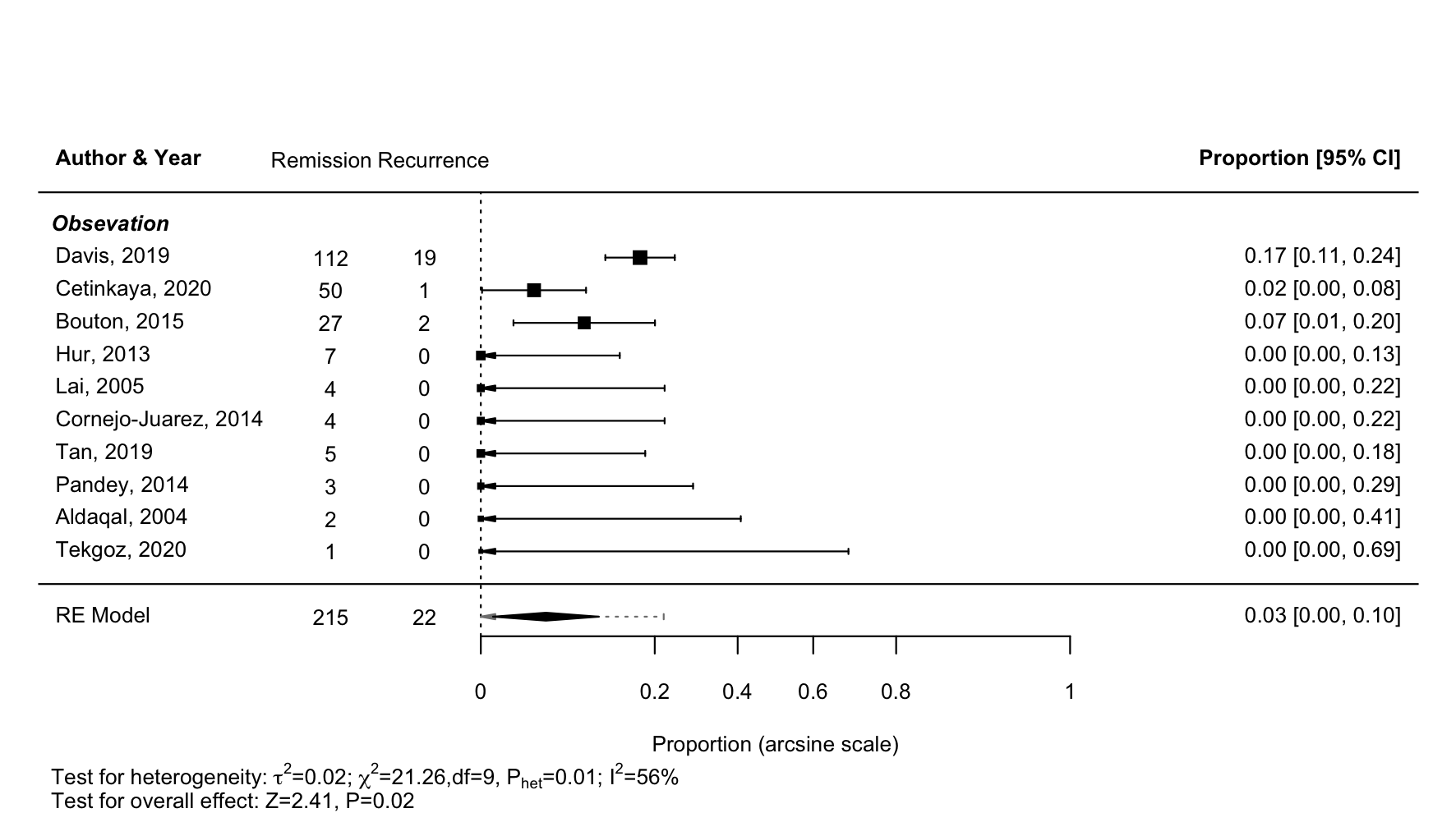

Supplement: Supplementary file 2 [file Presentation_1.ZIP › Supplementary Figure 1b.jpg]

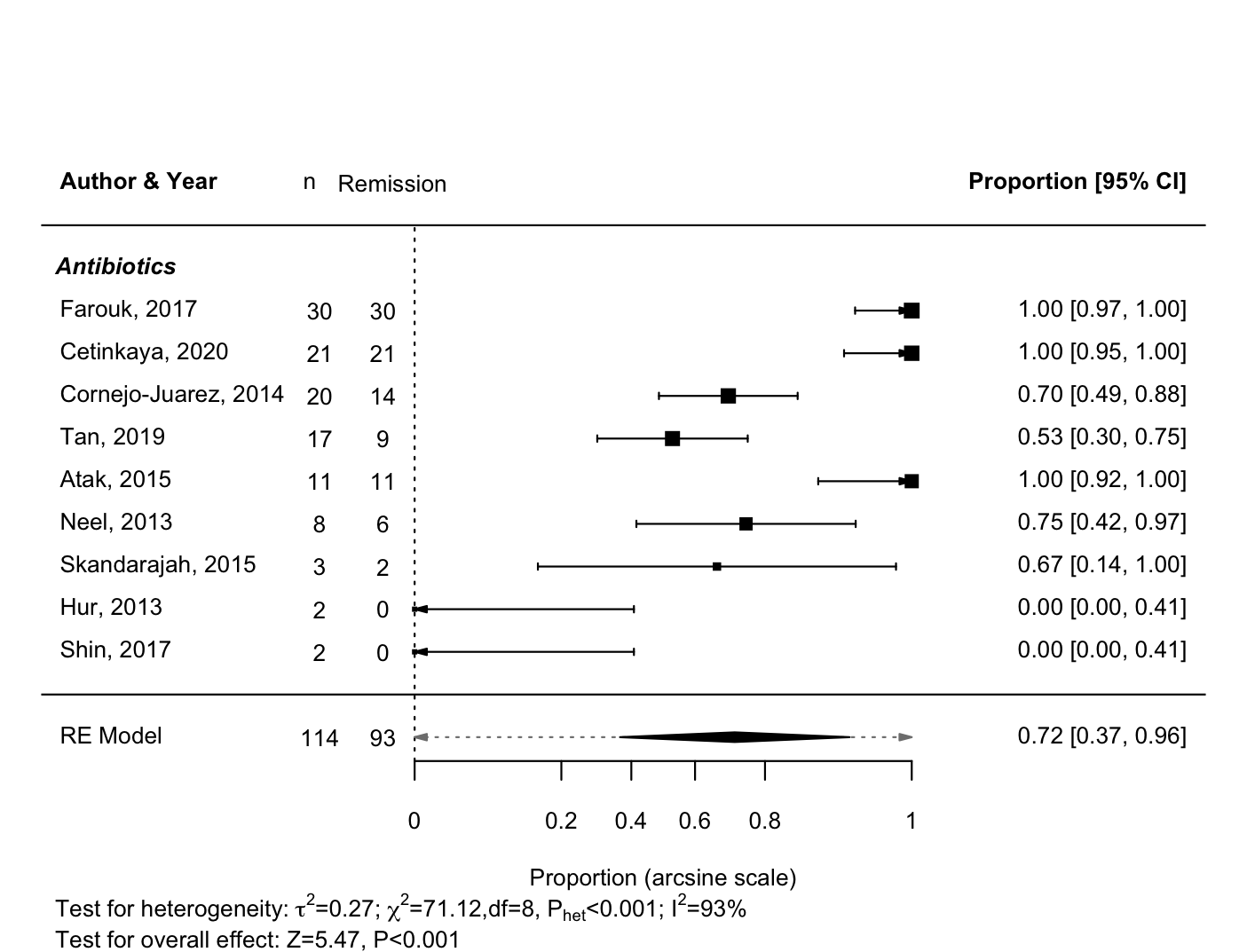

Supplement: Supplementary file 2 [file Presentation_1.ZIP › Supplementary Figure 2a.jpg]

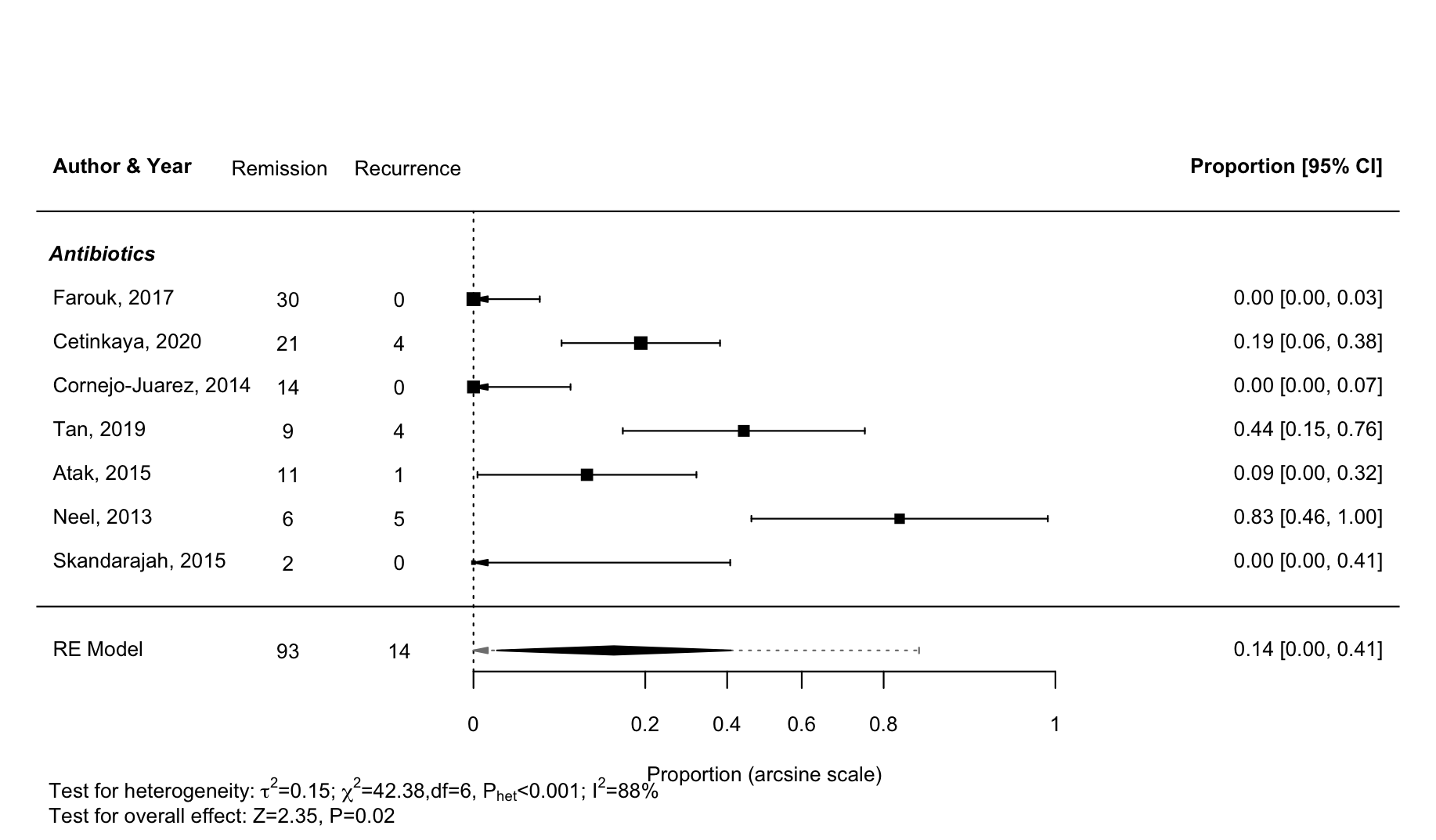

Supplement: Supplementary file 2 [file Presentation_1.ZIP › Supplementary Figure 2b.jpg]

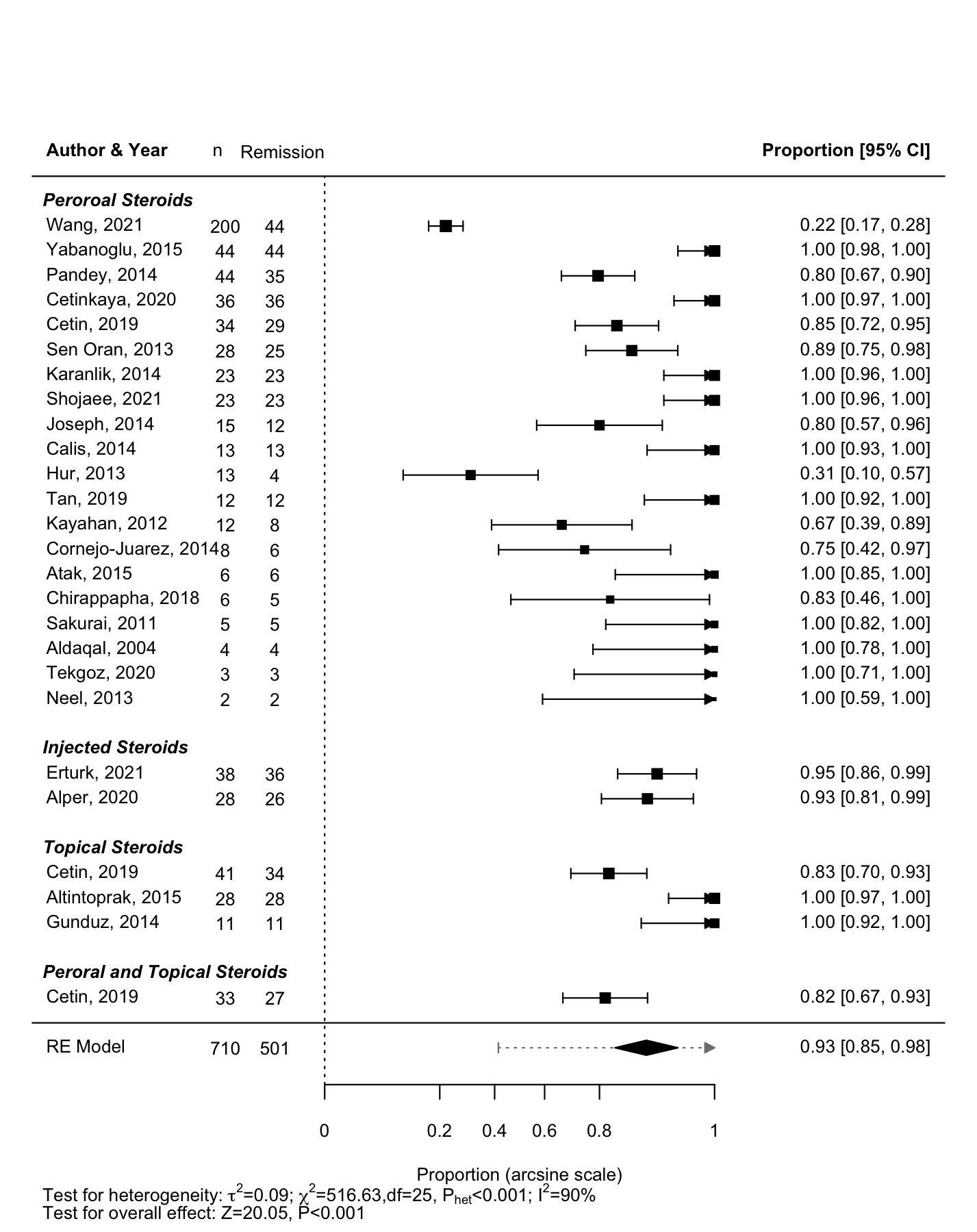

Supplement: Supplementary file 2 [file Presentation_1.ZIP › Supplementary Figure 3a.jpg]

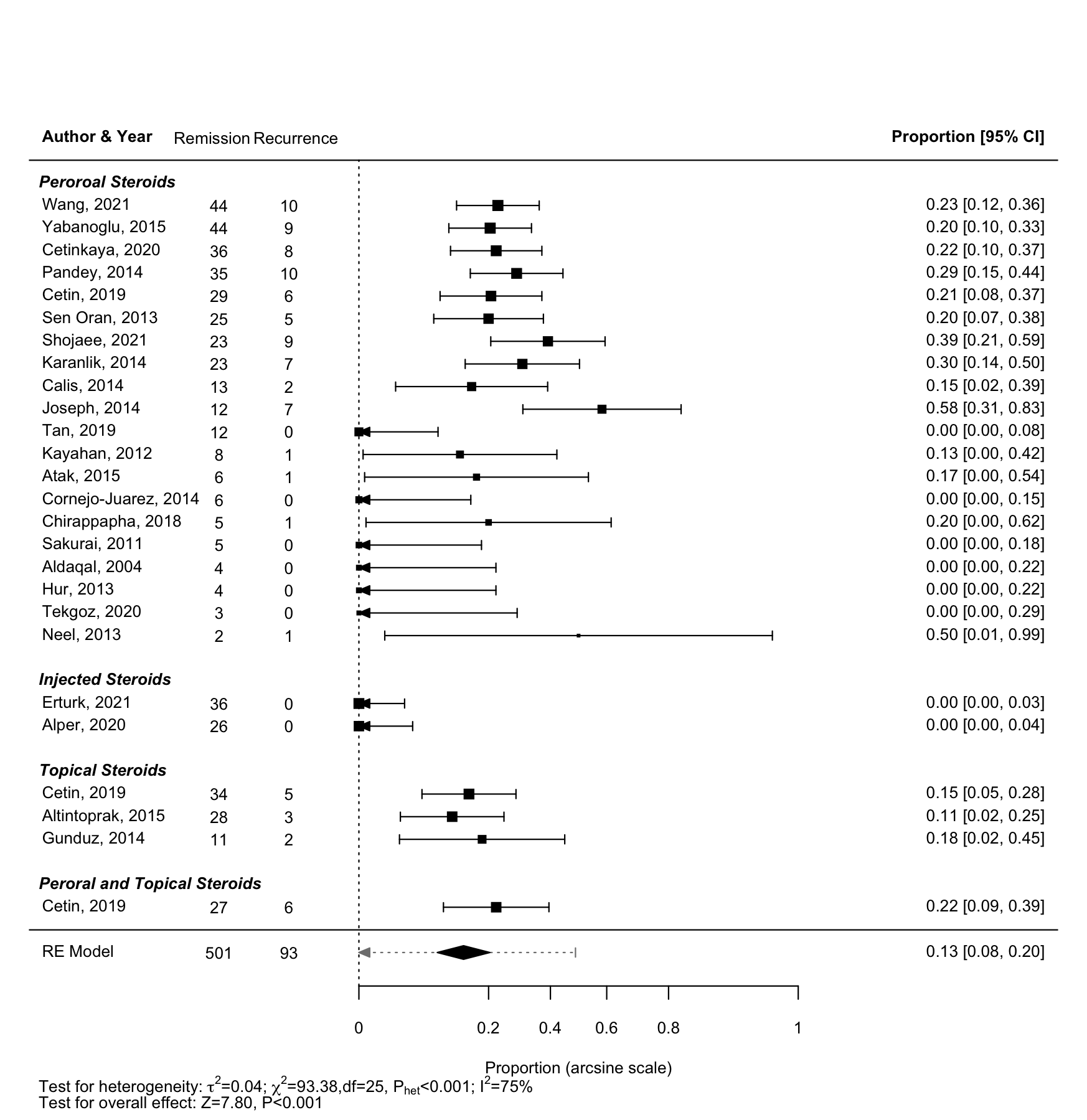

Supplement: Supplementary file 2 [file Presentation_1.ZIP › Supplementary Figure 3b.jpg]

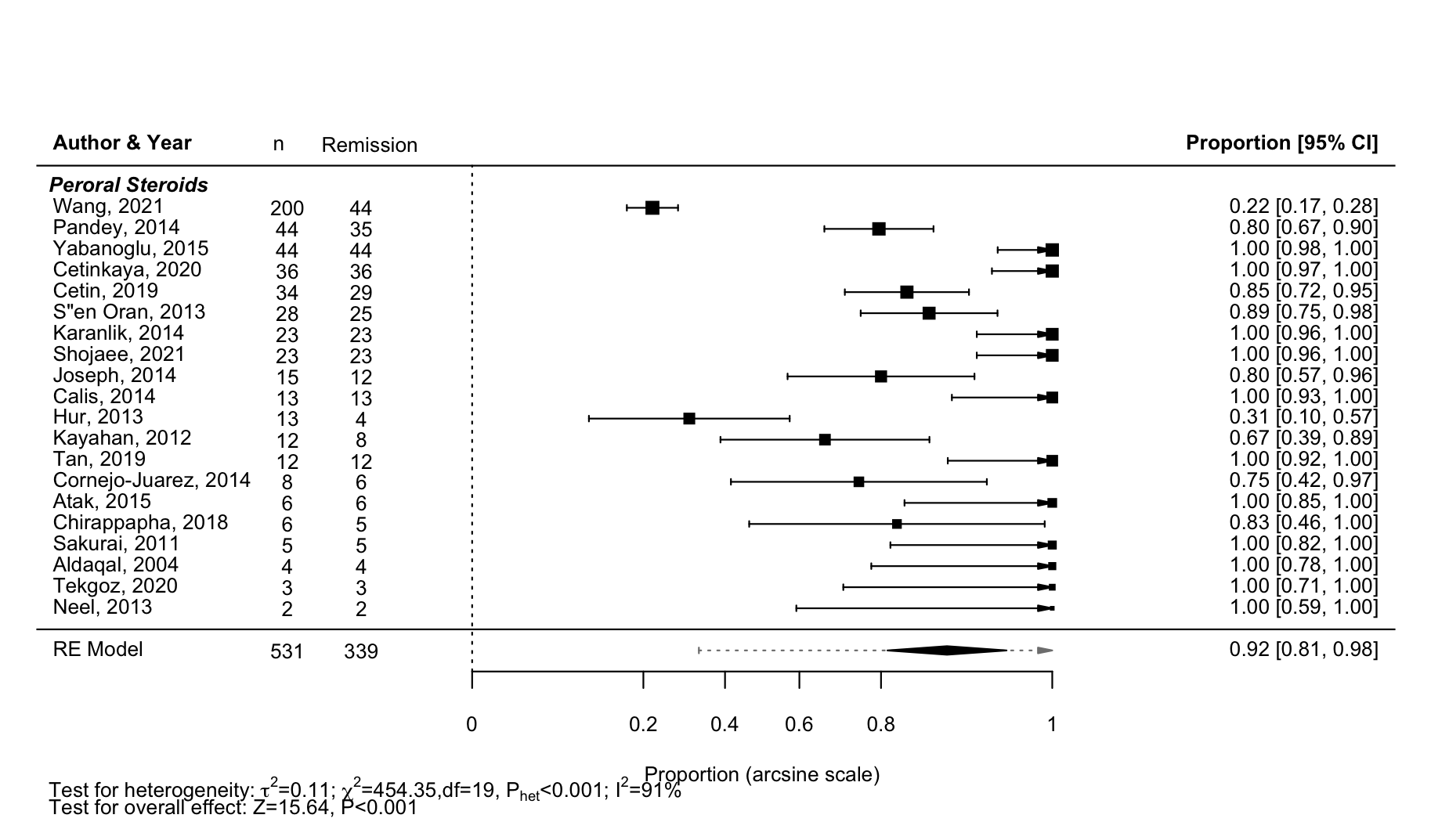

Supplement: Supplementary file 2 [file Presentation_1.ZIP › Supplementary Figure 3c.jpg]

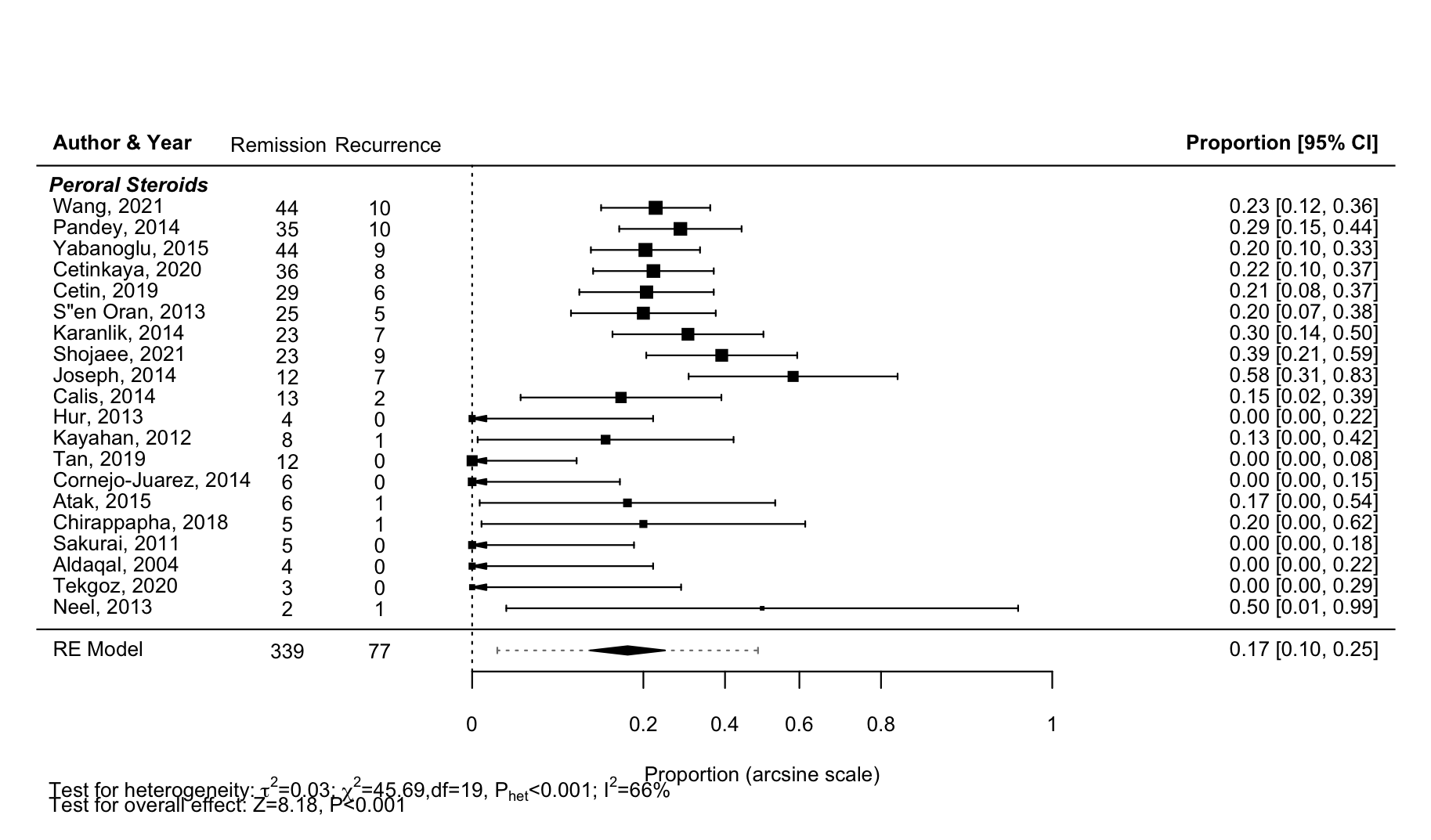

Supplement: Supplementary file 2 [file Presentation_1.ZIP › Supplementary Figure 3d.jpg]

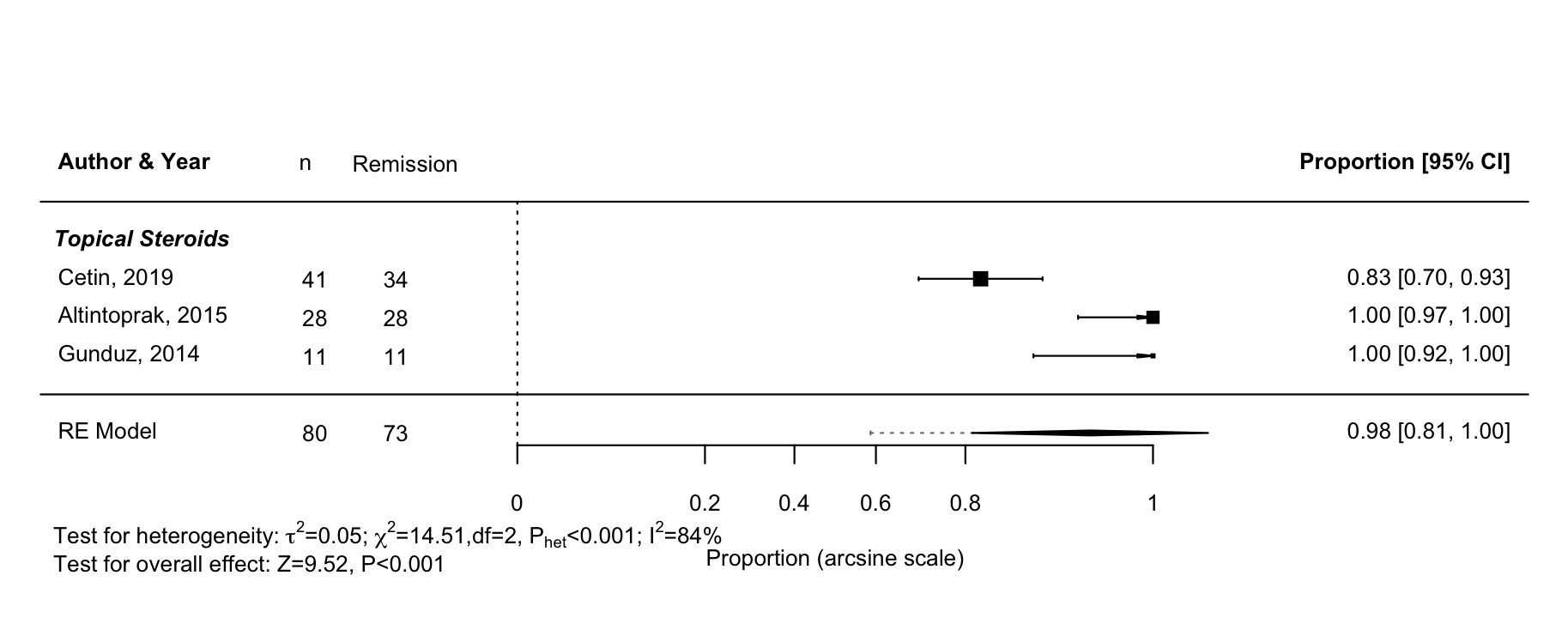

Supplement: Supplementary file 2 [file Presentation_1.ZIP › Supplementary Figure 3e.jpg]

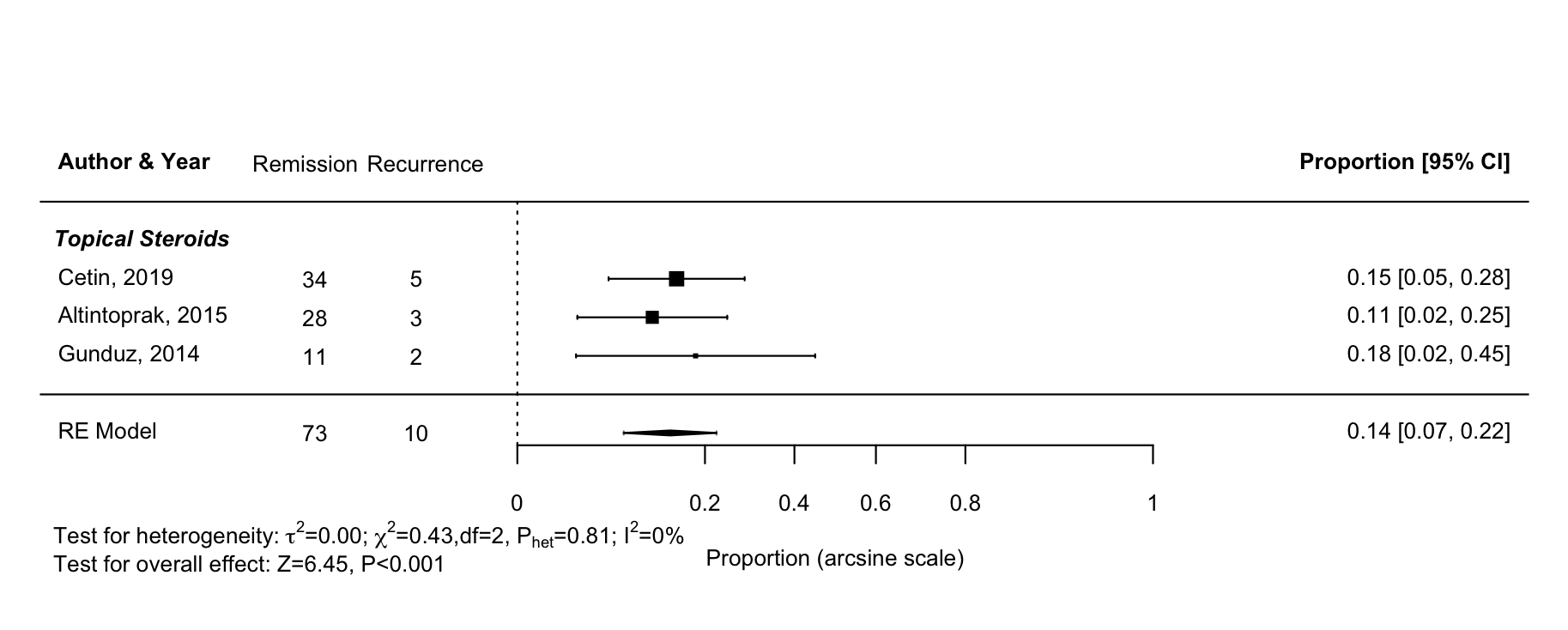

Supplement: Supplementary file 2 [file Presentation_1.ZIP › Supplementary Figure 3f.jpg]

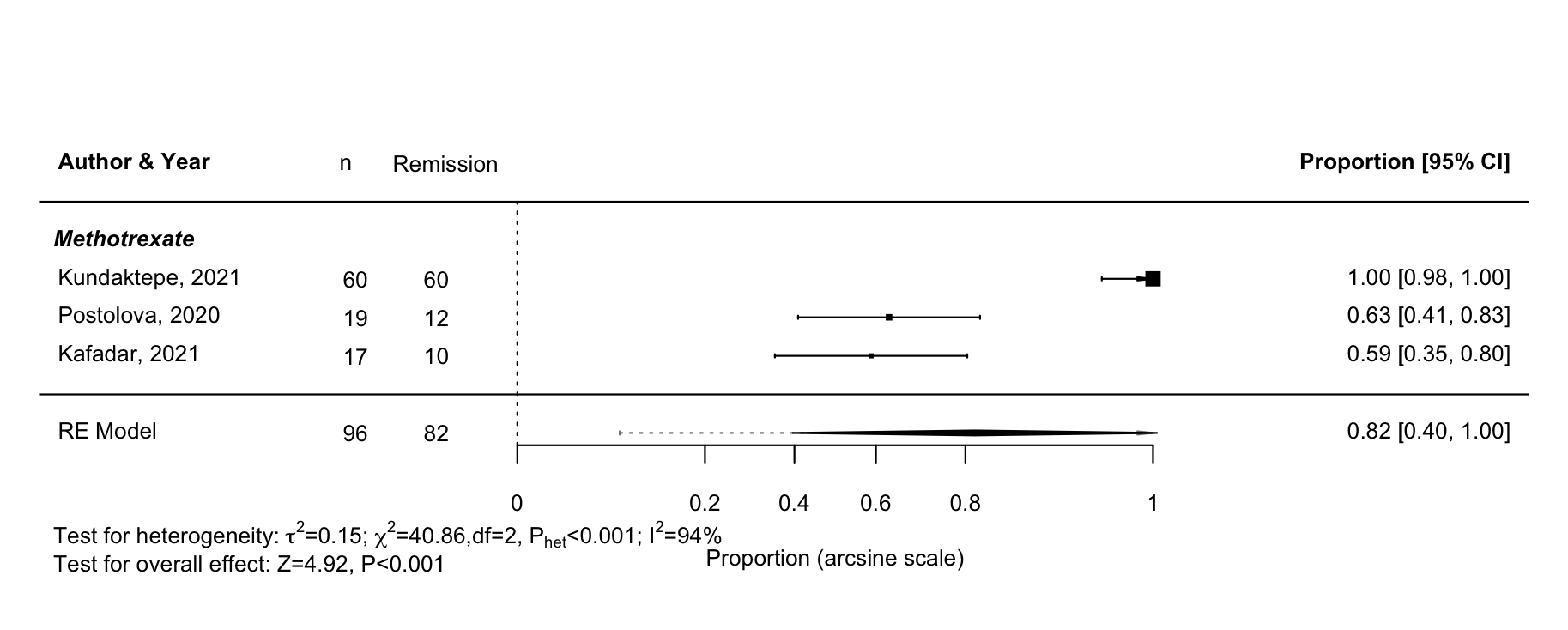

Supplement: Supplementary file 2 [file Presentation_1.ZIP › Supplementary Figure 4a.jpg]

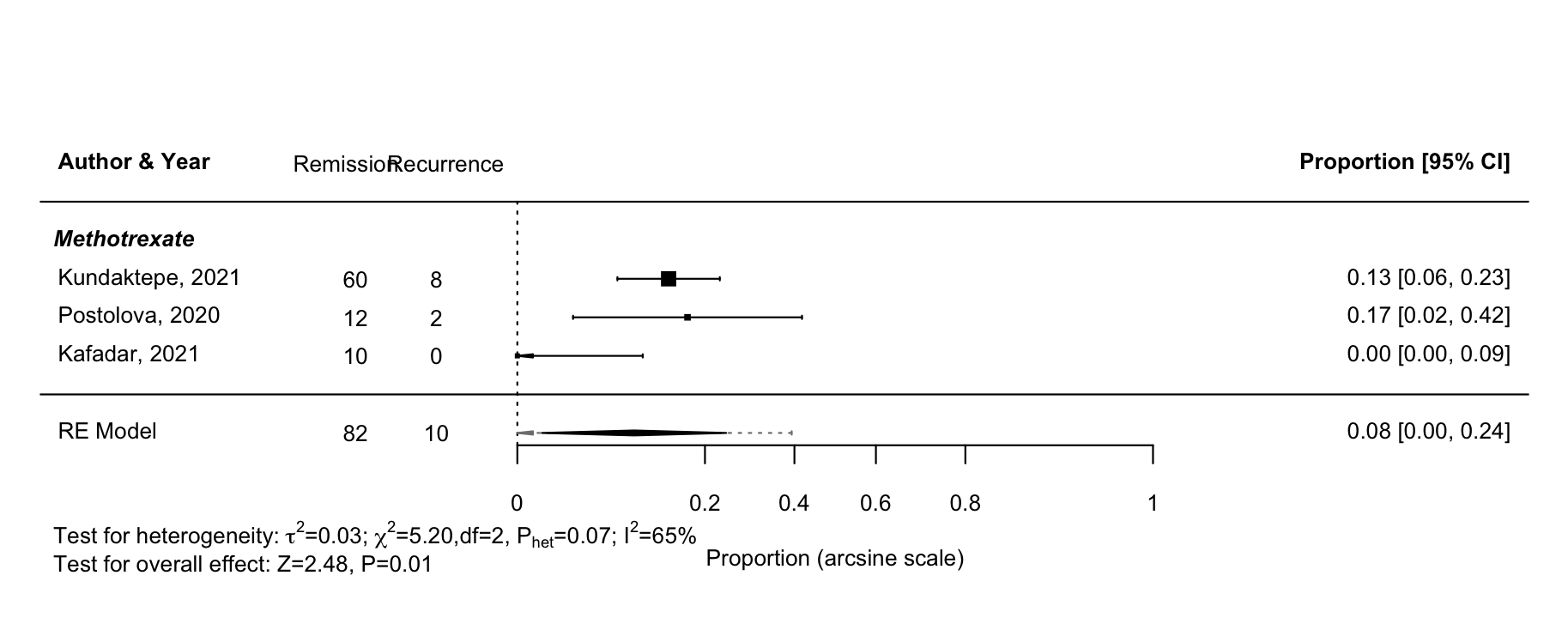

Supplement: Supplementary file 2 [file Presentation_1.ZIP › Supplementary Figure 4b.jpg]

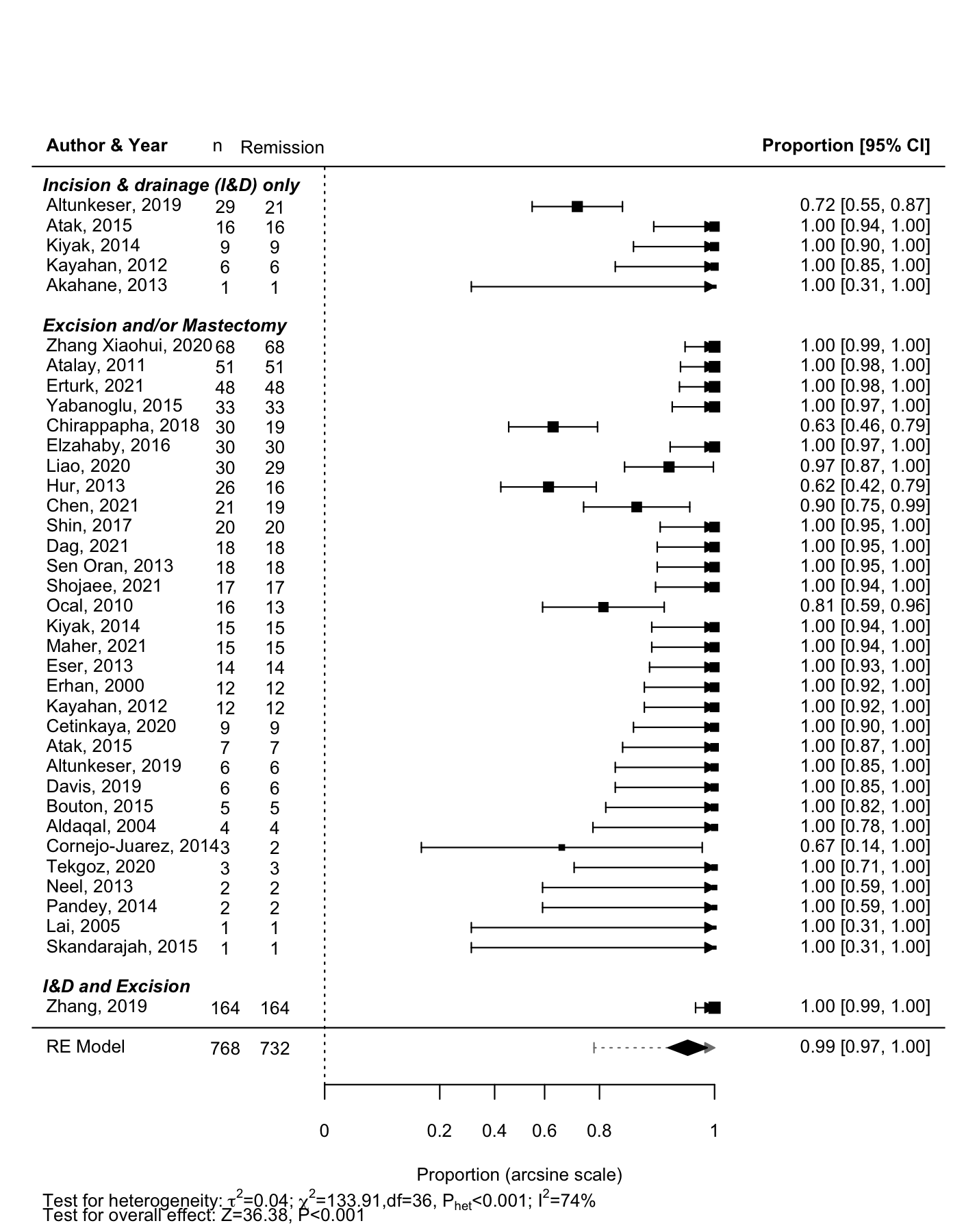

Supplement: Supplementary file 2 [file Presentation_1.ZIP › Supplementary Figure 5a.jpg]

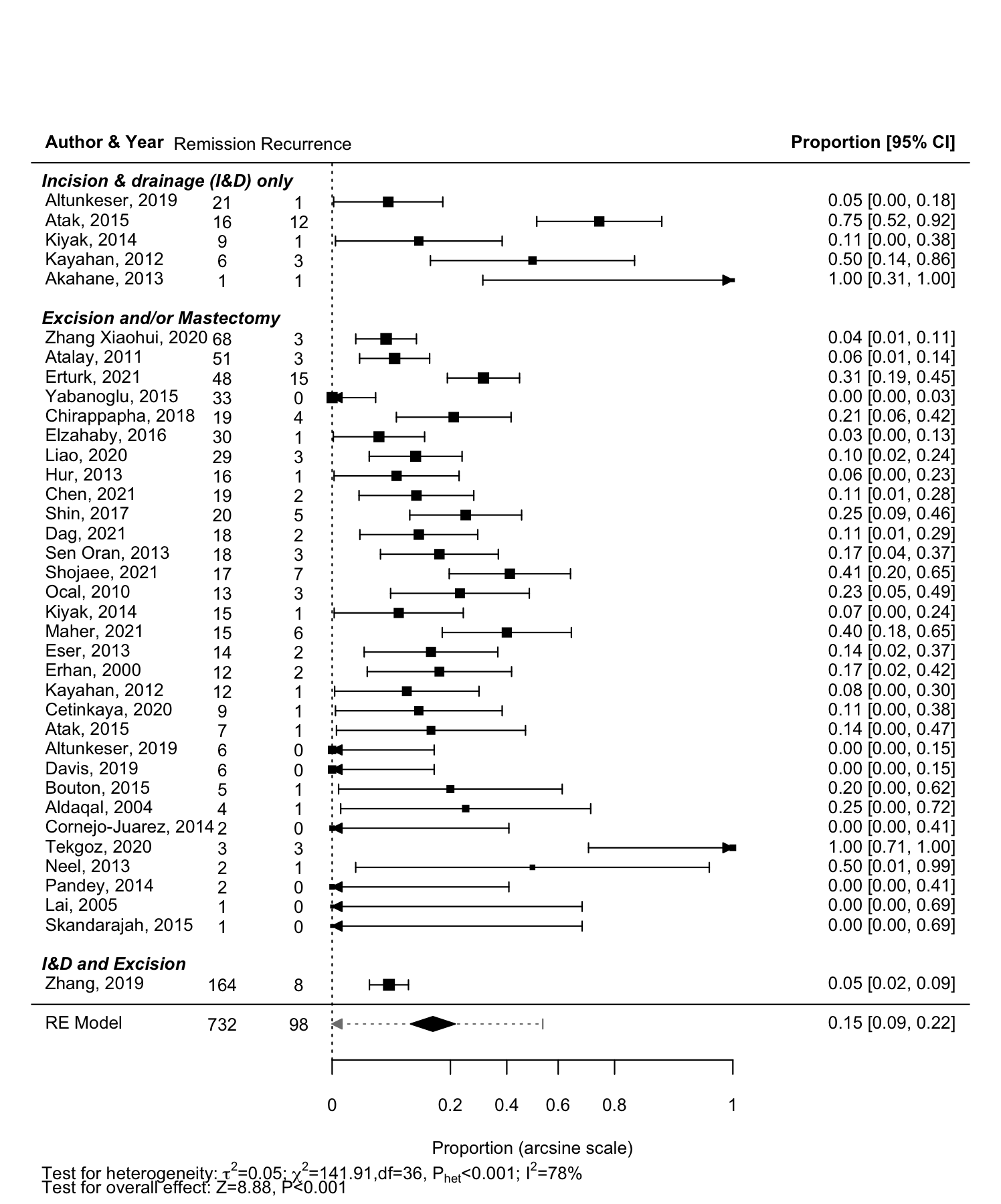

Supplement: Supplementary file 2 [file Presentation_1.ZIP › Supplementary Figure 5b.jpg]

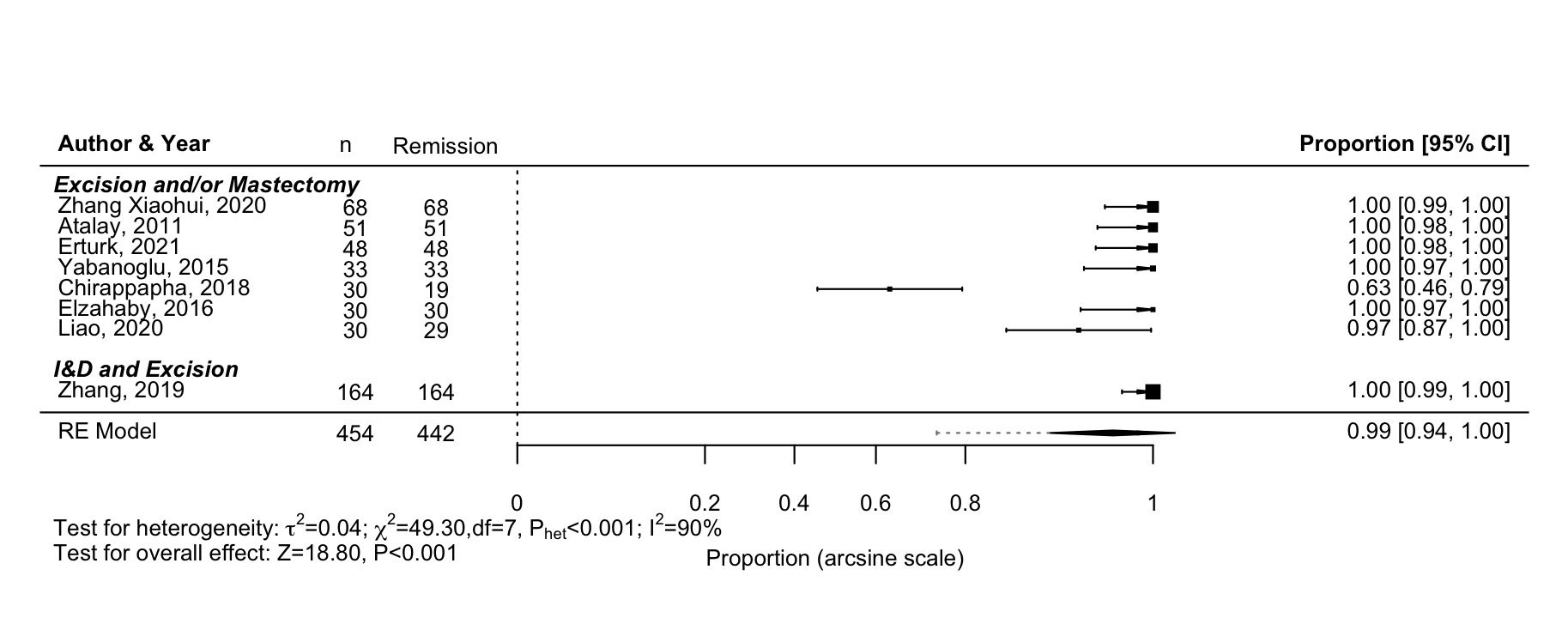

Supplement: Supplementary file 2 [file Presentation_1.ZIP › Supplementary Figure 5c.jpg]

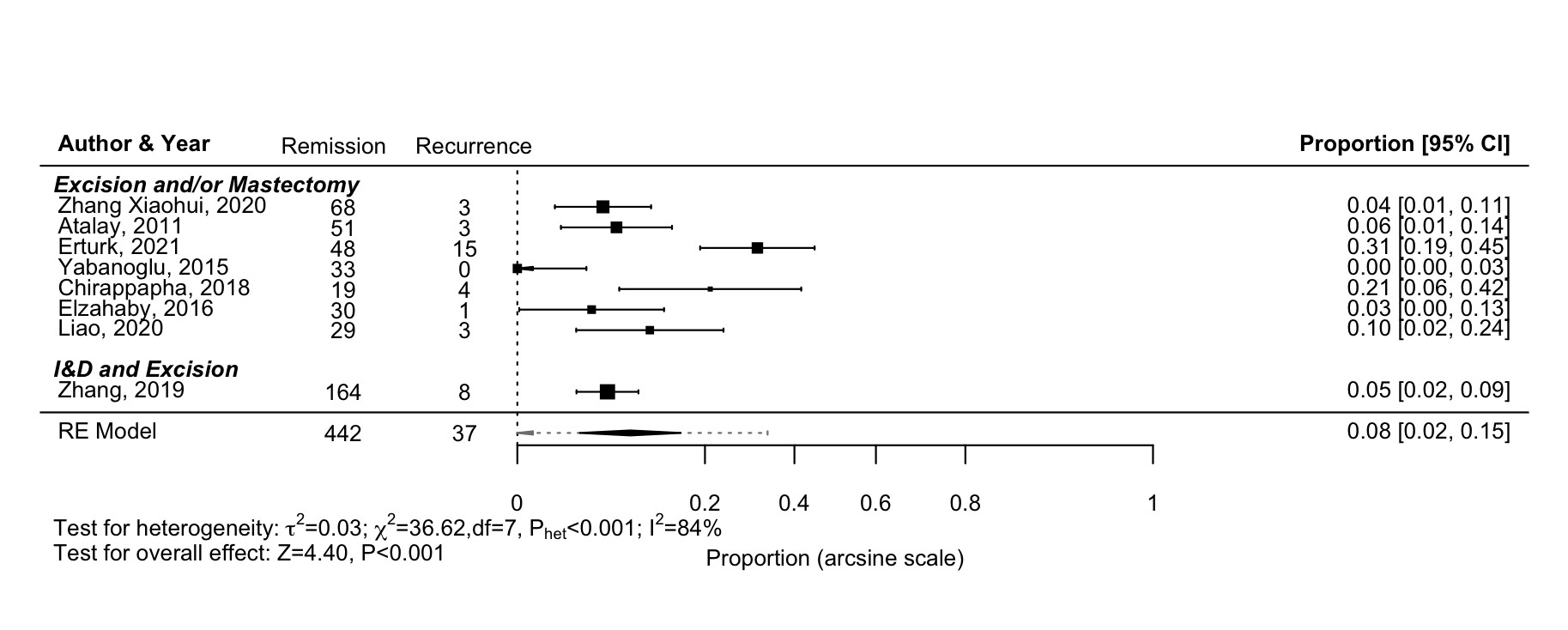

Supplement: Supplementary file 2 [file Presentation_1.ZIP › Supplementary Figure 5d.jpg]

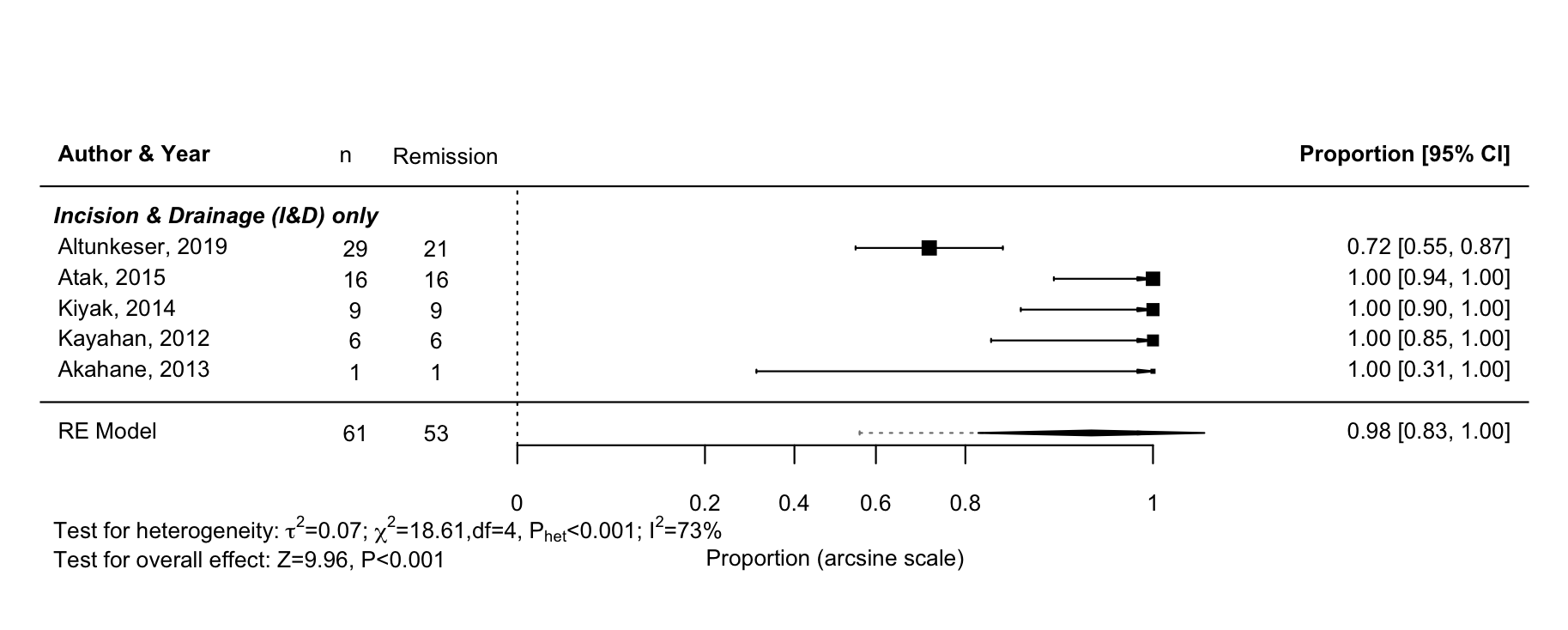

Supplement: Supplementary file 2 [file Presentation_1.ZIP › Supplementary Figure 5e.jpg]

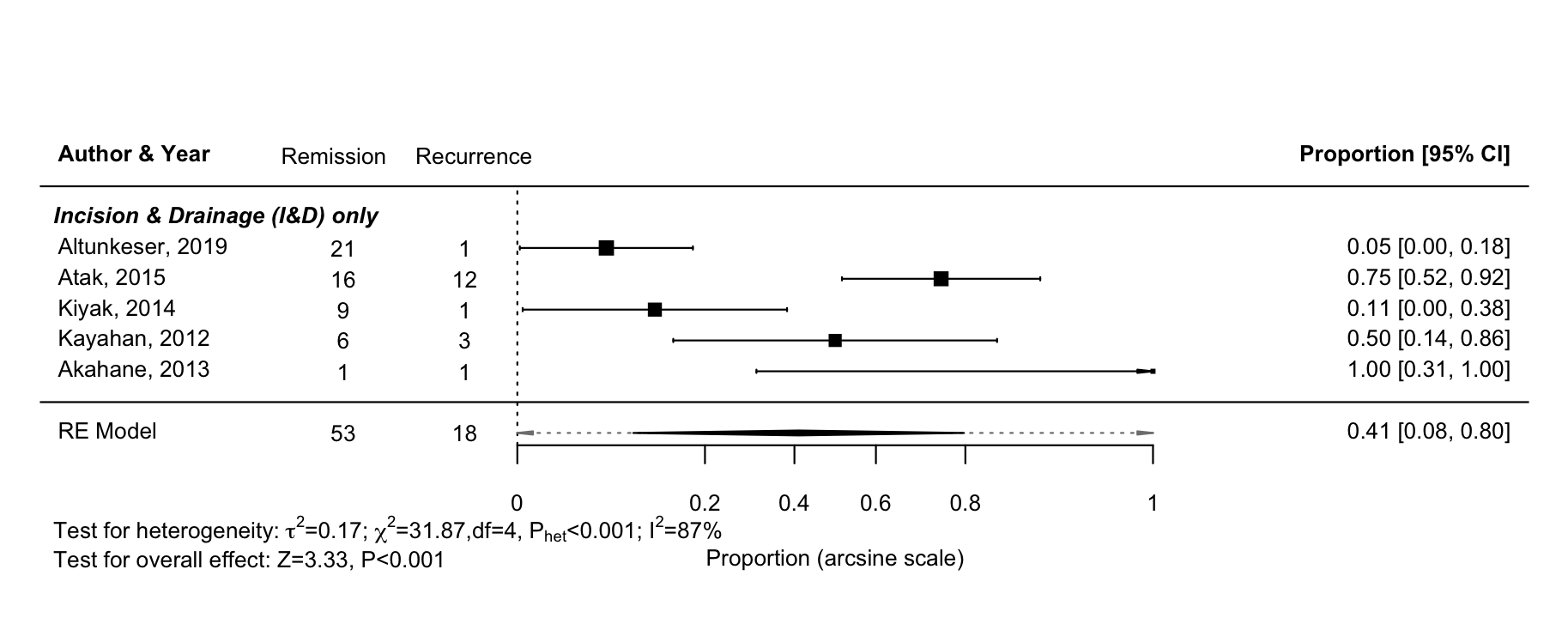

Supplement: Supplementary file 2 [file Presentation_1.ZIP › Supplementary Figure 5f.jpg]

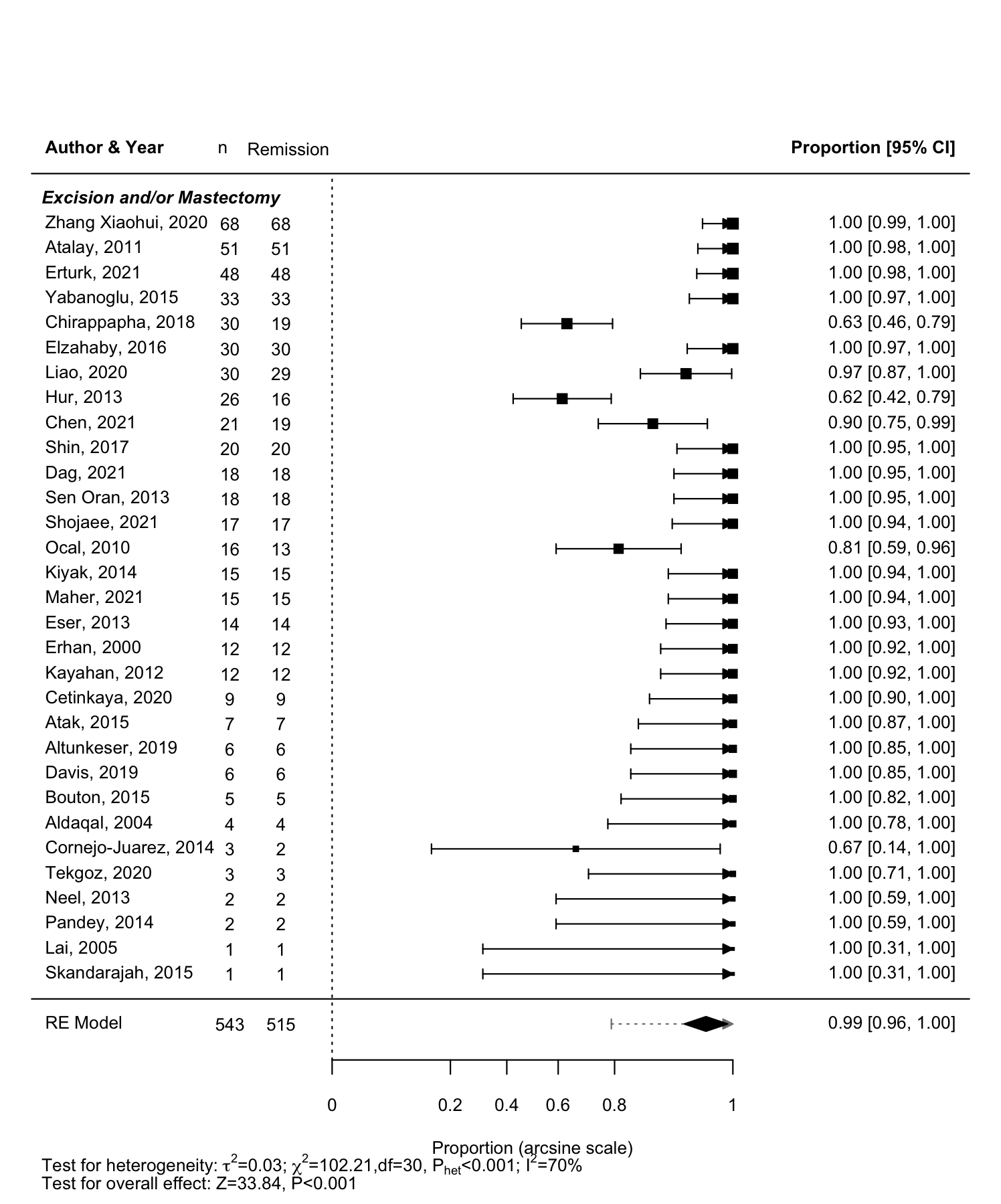

Supplement: Supplementary file 2 [file Presentation_1.ZIP › Supplementary Figure 5g.jpg]

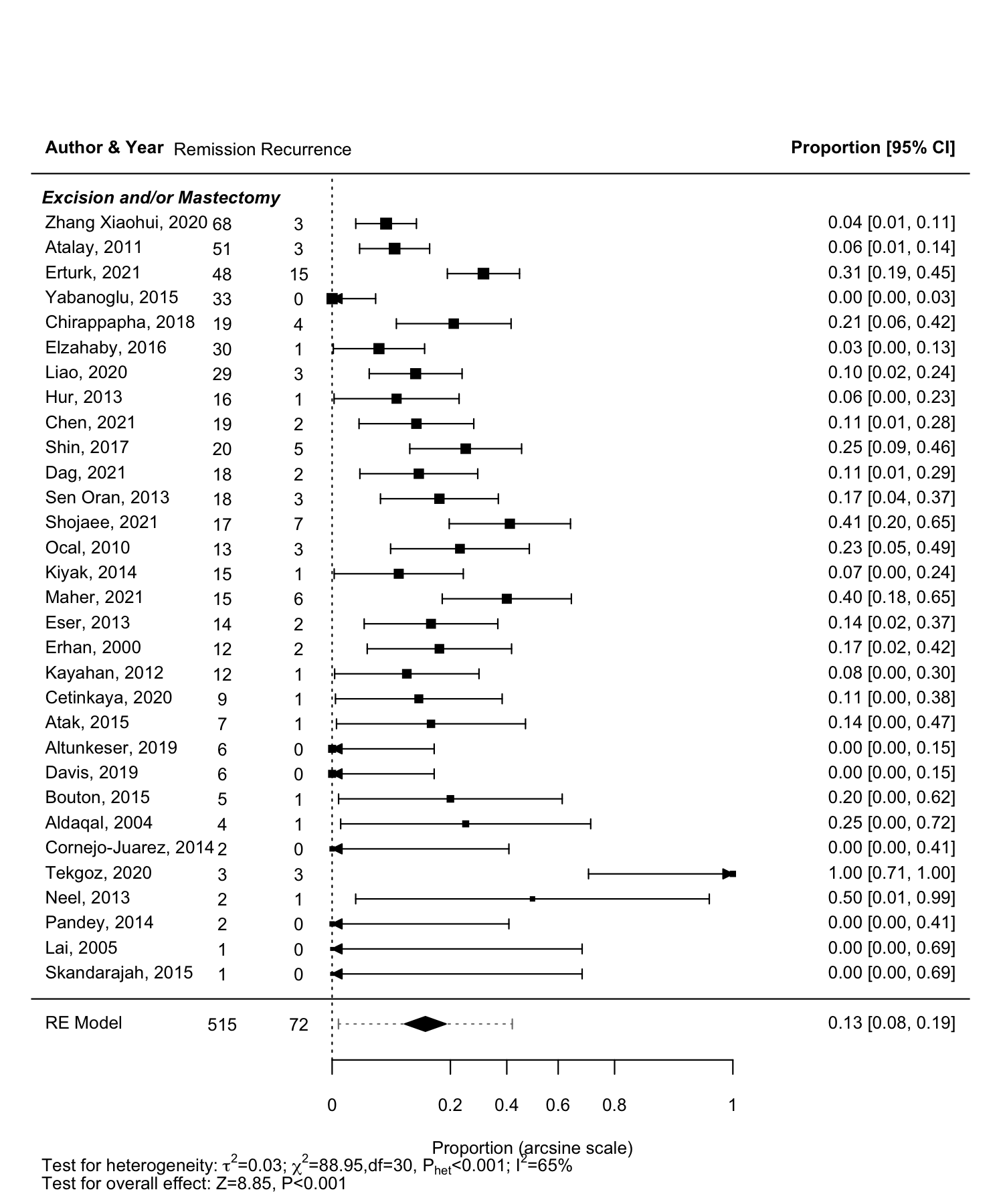

Supplement: Supplementary file 2 [file Presentation_1.ZIP › Supplementary Figure 5h.jpg]

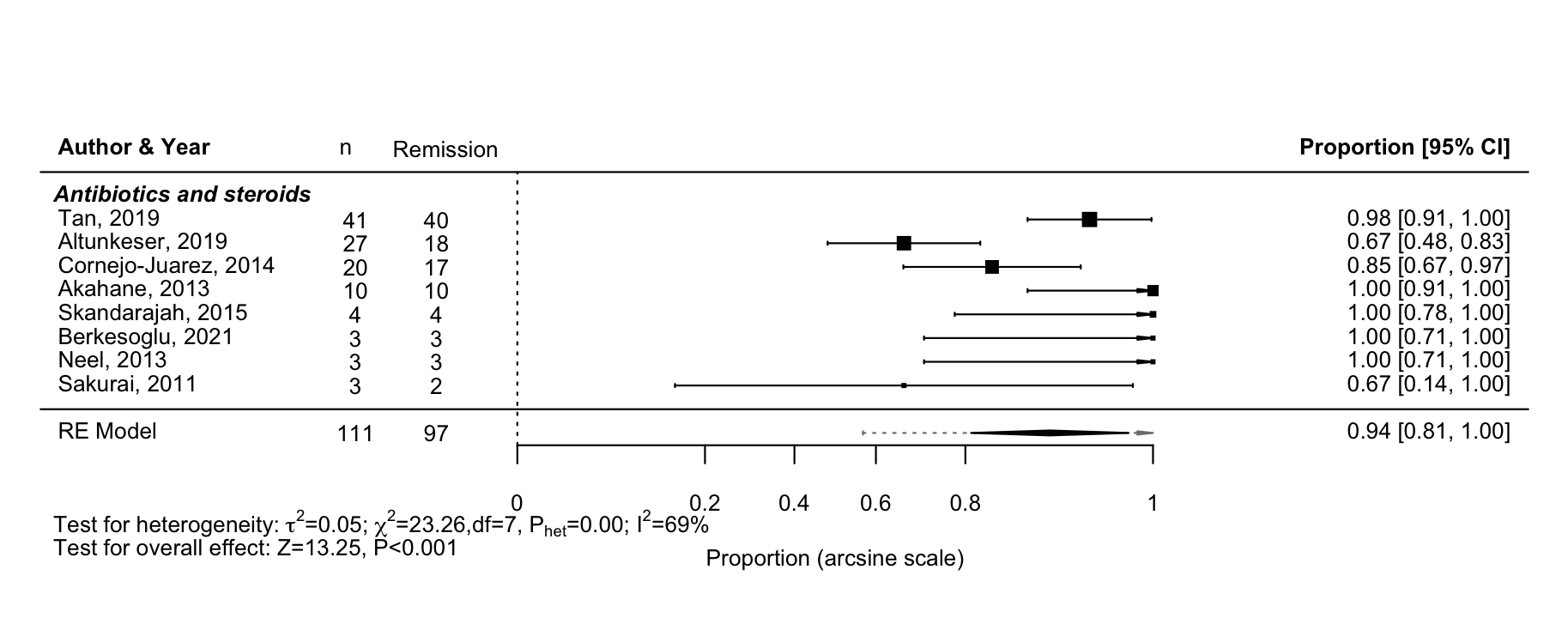

Supplement: Supplementary file 2 [file Presentation_1.ZIP › Supplementary Figure 6a.jpg]

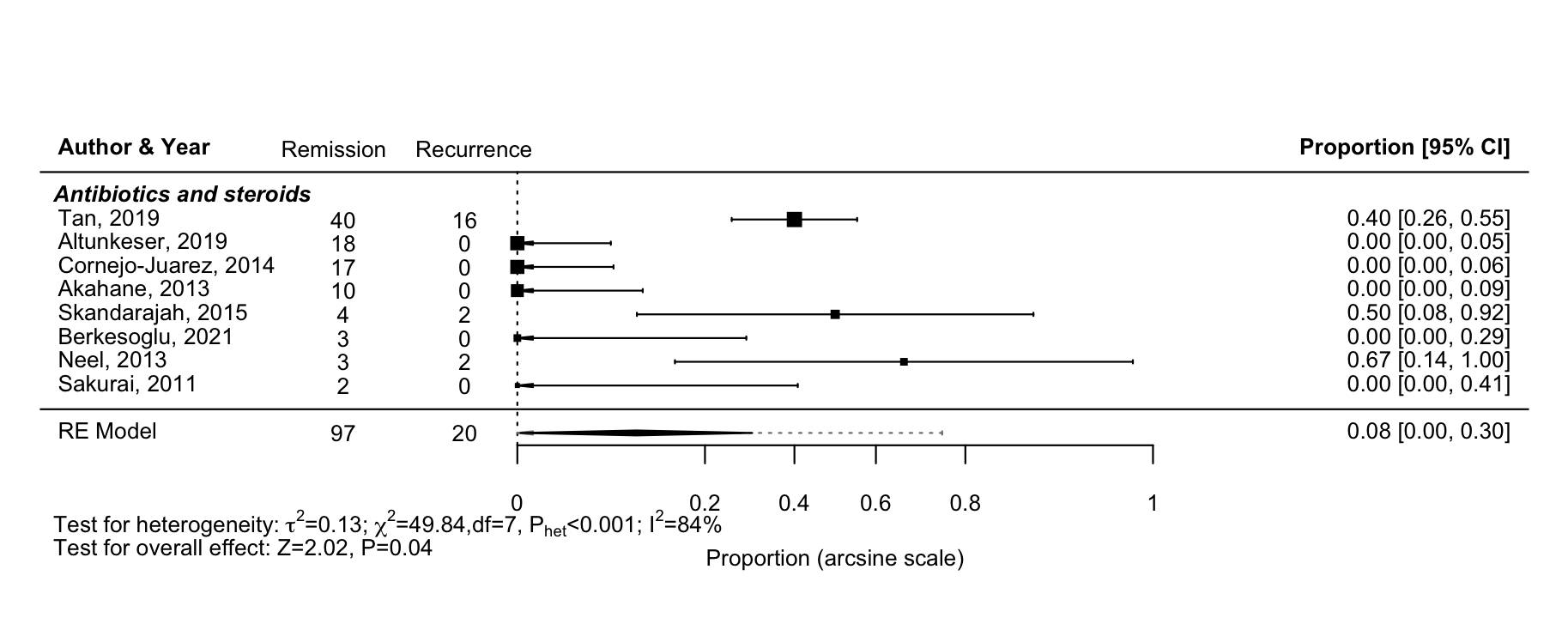

Supplement: Supplementary file 2 [file Presentation_1.ZIP › Supplementary Figure 6b.jpg]

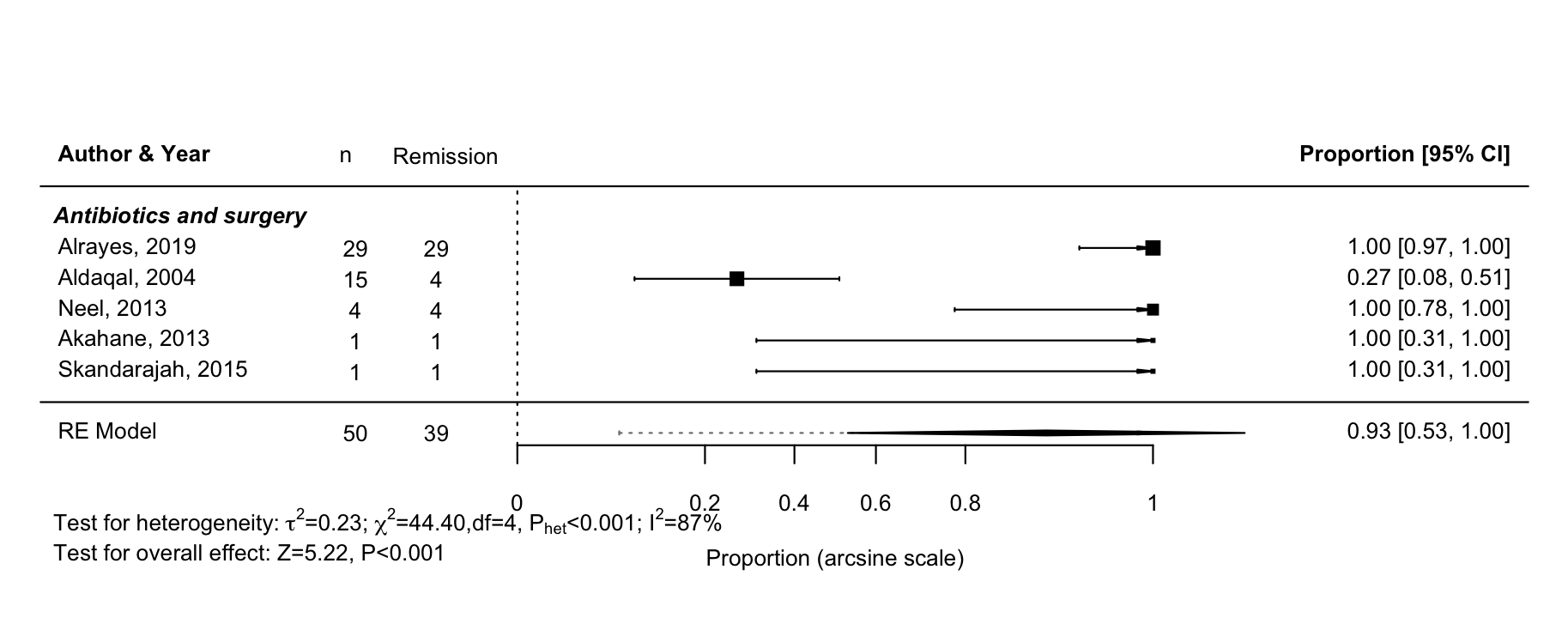

Supplement: Supplementary file 2 [file Presentation_1.ZIP › Supplementary Figure 6c.jpg]

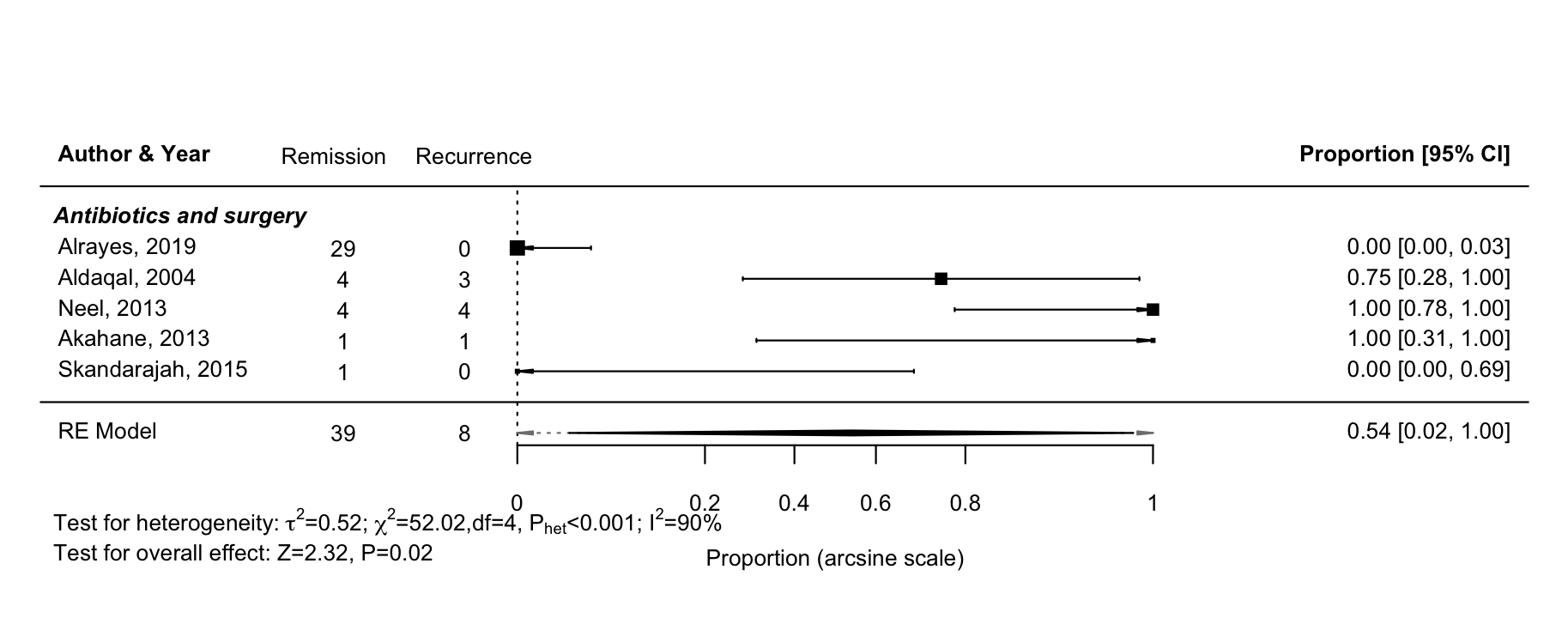

Supplement: Supplementary file 2 [file Presentation_1.ZIP › Supplementary Figure 6d.jpg]

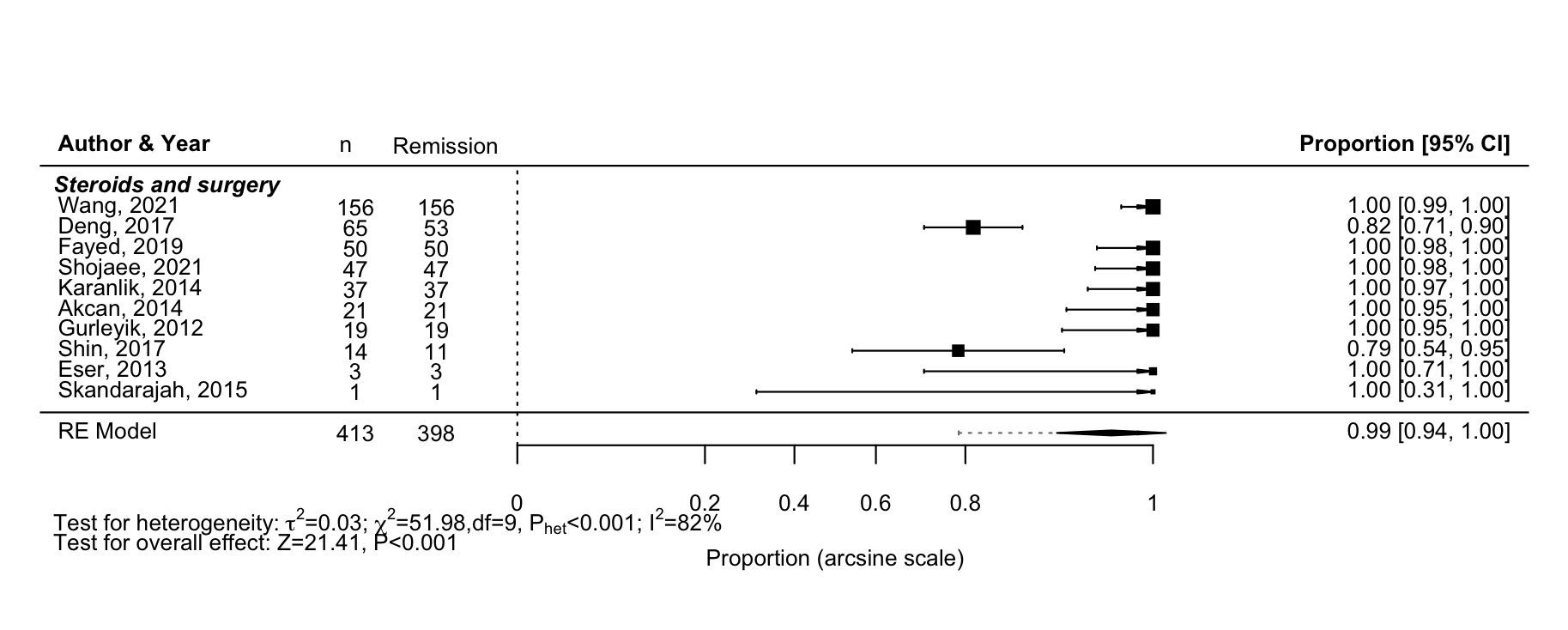

Supplement: Supplementary file 2 [file Presentation_1.ZIP › Supplementary Figure 6e.jpg]

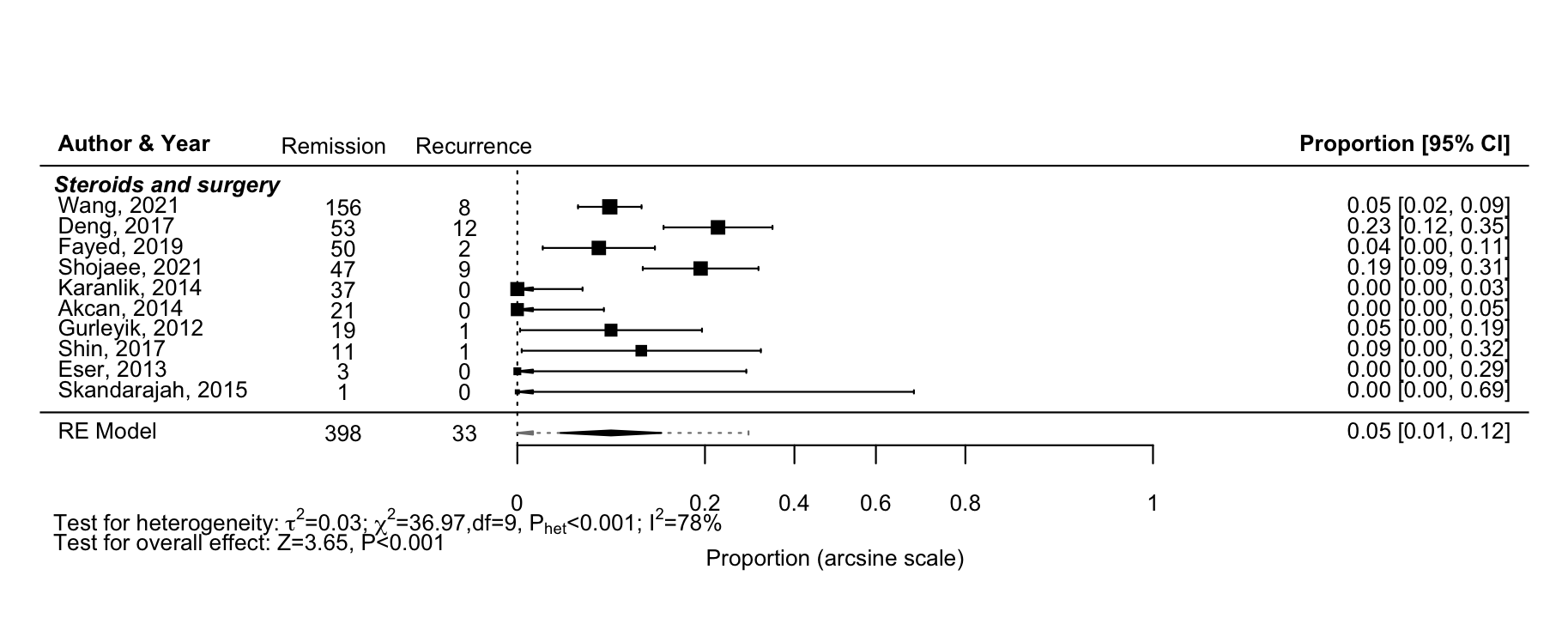

Supplement: Supplementary file 2 [file Presentation_1.ZIP › Supplementary Figure 6f.jpg]

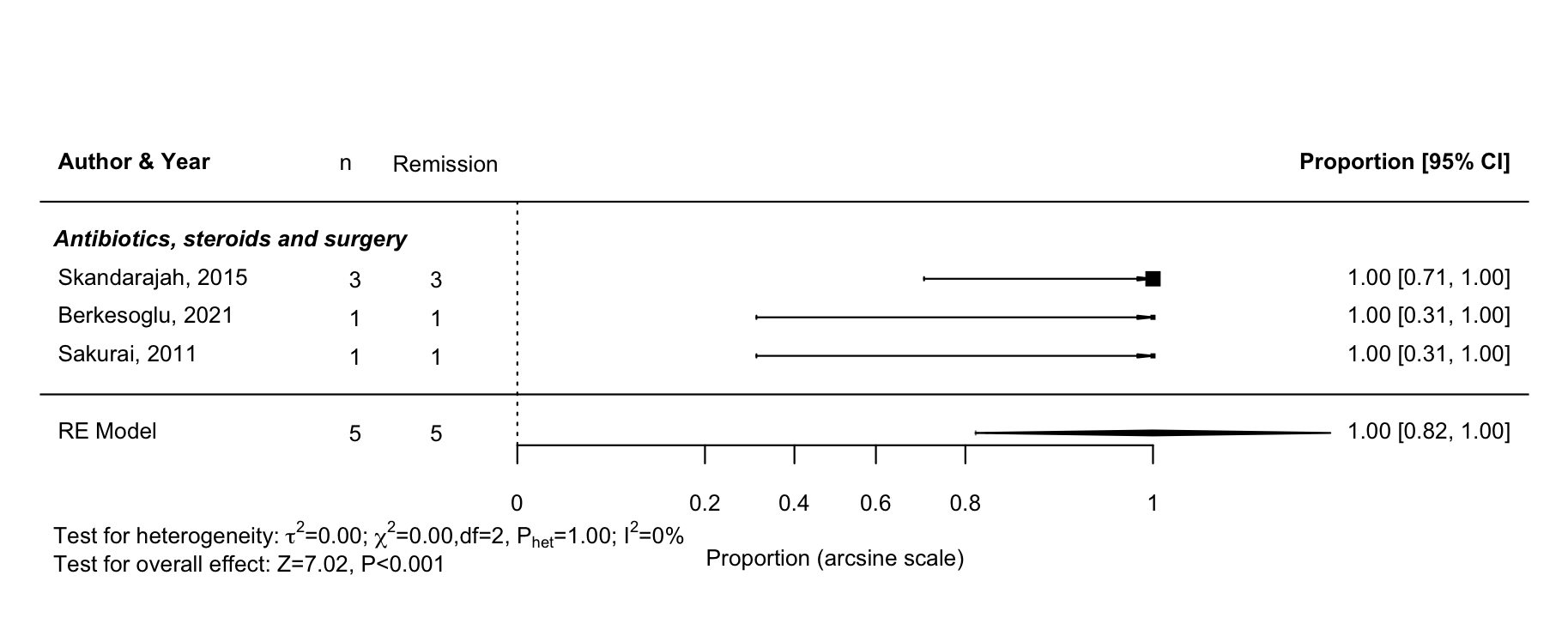

Supplement: Supplementary file 2 [file Presentation_1.ZIP › Supplementary Figure 6g.jpg]

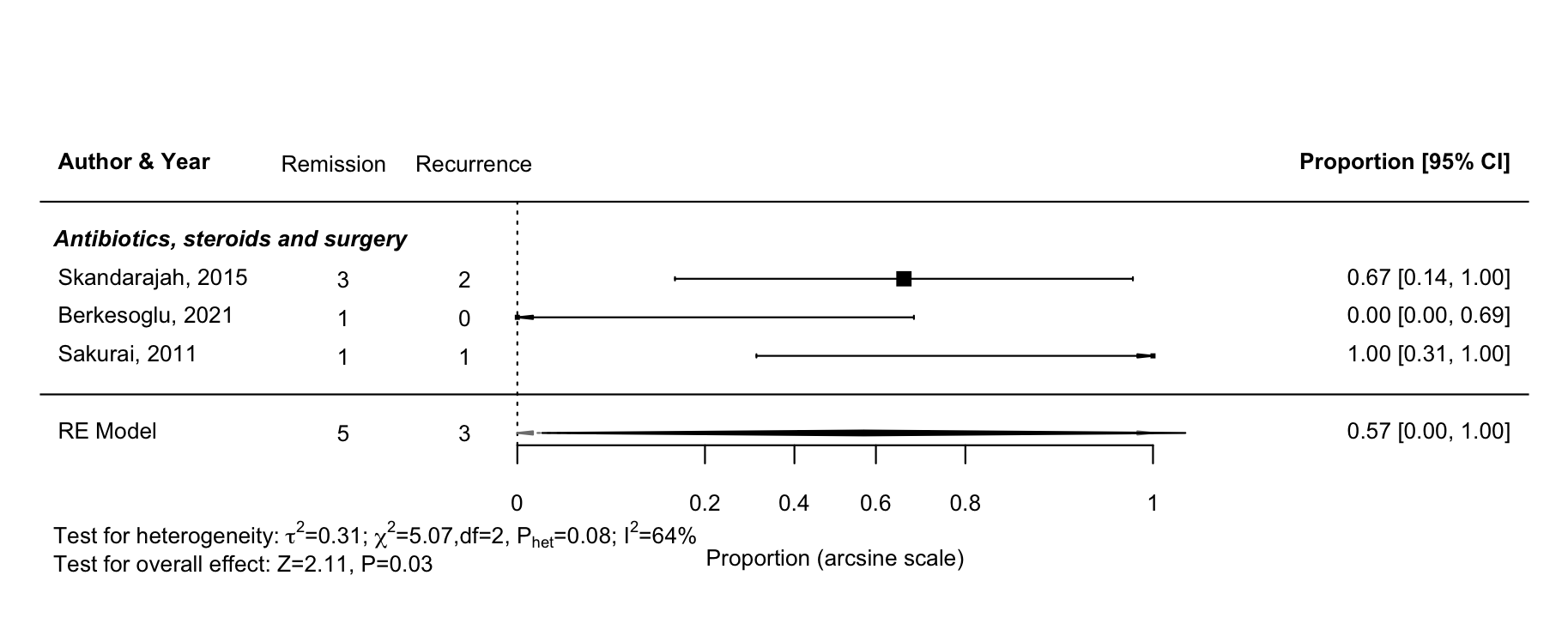

Supplement: Supplementary file 2 [file Presentation_1.ZIP › Supplementary Figure 6h.jpg]

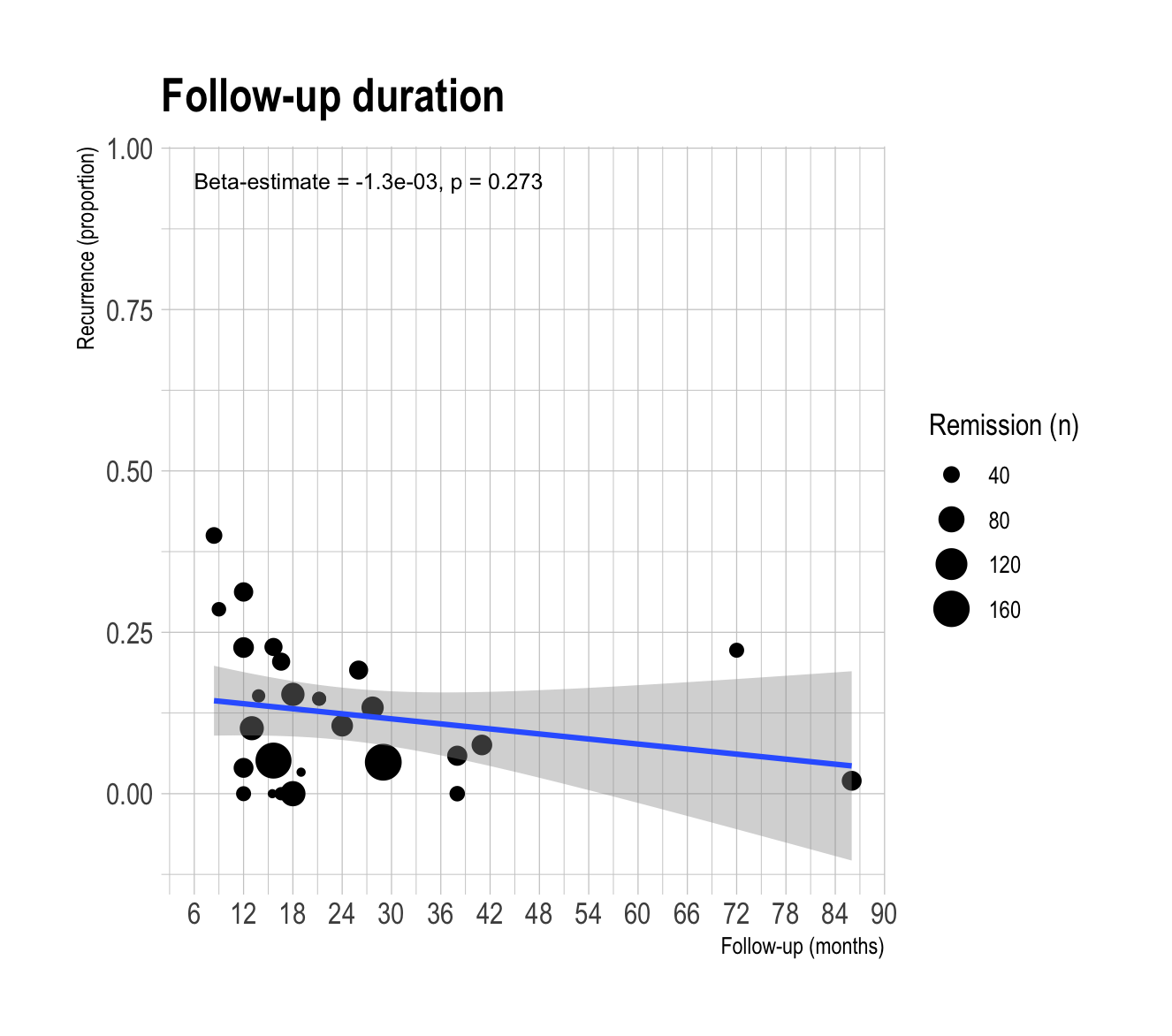

Supplement: Supplementary file 2 [file Presentation_1.ZIP › Supplementary Figure 7.jpg]
